# Recent Advances of Chitosan Formulations in Biomedical Applications

**DOI:** 10.3390/ijms231810975

**Published:** 2022-09-19

**Authors:** Mohammed A. S. Abourehab, Sheersha Pramanik, Mohamed A. Abdelgawad, Bassam M. Abualsoud, Ammar Kadi, Mohammad Javed Ansari, A. Deepak

**Affiliations:** 1 Department of Pharmaceutics, College of Pharmacy, Umm Al Qura University, Makkah 21955, Saudi Arabia; 2 Department of Pharmaceutics and Industrial Pharmacy, Faculty of Pharmacy, Minia University, Minia 11566, Egypt; 3 Department of Biotechnology, Bhupat and Jyoti Mehta School of Biosciences, Indian Institute of Technology Madras, Chennai 600036, India; 4 Department of Pharmaceutical Chemistry, College of Pharmacy, Jouf University, Sakaka 72341, Saudi Arabia; 5 Department of Pharmaceutics and Pharmaceutical Technology, College of Pharmacy, Al-Ahliyya Amman University, Amman 19328, Jordan; 6 Department of Food and Biotechnology, South Ural State University, 454080 Chelyabinsk, Russia; 7 Department of Pharmaceutics, College of Pharmacy, Prince Sattam Bin Abdulaziz University, Al-Kharj 11942, Saudi Arabia; 8 Saveetha School of Engineering, Saveetha Institute of Medical and Technical Sciences, Chennai 600128, India

**Keywords:** chitosan, natural polymer, biomedical applications, drug delivery, tissue engineering, wound healing

## Abstract

Chitosan, a naturally abundant cationic polymer, is chemically composed of cellulose-based biopolymers derived by deacetylating chitin. It offers several attractive characteristics such as renewability, hydrophilicity, biodegradability, biocompatibility, non-toxicity, and a broad spectrum of antimicrobial activity towards gram-positive and gram-negative bacteria as well as fungi, etc., because of which it is receiving immense attention as a biopolymer for a plethora of applications including drug delivery, protective coating materials, food packaging films, wastewater treatment, and so on. Additionally, its structure carries reactive functional groups that enable several reactions and electrochemical interactions at the biomolecular level and improves the chitosan’s physicochemical properties and functionality. This review article highlights the extensive research about the properties, extraction techniques, and recent developments of chitosan-based composites for drug, gene, protein, and vaccine delivery applications. Its versatile applications in tissue engineering and wound healing are also discussed. Finally, the challenges and future perspectives for chitosan in biomedical applications are elucidated.

## 1. Introduction

Carbohydrates, the most common natural polymers, join their monomeric units through glycosidic linkages. Some of the beneficial polysaccharides in the biomedical field include starch, cellulose, chitin, pectin, and so on [1]. Scientists in the polymeric field have regarded chitin and chitosan as important biopolymers in the biomedical, electronic, and pharmaceutical fields [2]. Representing one of the most abundant natural polymers, the polycationic biopolymer, chitosan, has several applications such as sewage purification [3], cell entrapment coacervation [4], and seed coating for higher crop yields [5], and also as a food packaging material [6].

Chitosan is a straight-chain polymer of a (1→4)-linked 2-amino-2-deoxy-D-glucopyranose with some residual D-glucosamine units, that can be readily obtained by N-deacetylation of the highly crystalline heteropolymer, chitin [7]. It is naturally found in the cell walls of filamentous fungi, particularly the *Zygomycetes* class [8]. It refers to a heterogenous collection of oligomers and polymers which are distinct in the various degrees of polymerization, portions of acetylation, and the arrangements of acetylation [9]. It is industrially manufactured by hydrolysis of the amino acetyl functional groups of chitins. Chitosan is more relevant industrially than chitin because of its reactive amino and hydroxyl groups, its low crystallinity which makes it more receptive to reagents, and its solubility in most organic acidic solutions below its pKa of 6.5 [10].

Chitosan is used in a variety of industries, including treating effluents (removal of metallic ions, dyes, and as a membrane in contaminant expulsion), the food manufacturing sector (fat binding and cholesterol-lowering, food additives, packaging, and preservatives, farming (seed and fertilizer coatings, controlled agrochemical discharge), paper manufacturing (surface treatment, adhesive paper), cosmetic products (skincare products, face creams, etc.), tissue regeneration, and wound repair [11]. Gels, nanofibers, membranes, beads, microparticles, nanoparticles, sponges, and scaffolds could all be manufactured readily from chitin and chitosan [12].

Among the different biopolymers available in nature, chitosan especially has garnered attention because of its unique properties, such as inherent antimicrobial properties, natural abundance, versatility, non-toxicity, and biodegradability. Its degradation product consists of an innocuous amino sugar that could be absorbed by human tissues. The availability of reactive functional groups on the chitosan backbone makes it convenient to tailor it using physical or chemical means to fabricate desirable scaffolds for biological purposes [13,14]. For example, chitosan exhibits functional properties such as mucoadhesion, colon targeting, efflux pump inhibition, permeation improvement, in situ gelation, transfection, and other properties owing to its amino functionality in the chitosan structure [15].

There has been a rise in the number of publications reviewing the various facets of the extraction, preparation, properties, and applications of chitosan in recent years [14,16,17,18,19]. This review highlights the most current and significant advances in utilizing chitosan and its nanocarriers as drug delivery systems, tissue engineering scaffolds, wound dressings, and vaccine delivery carriers. The scope of the present work is to highlight the various aspects of chitosan and its derivatives, sometimes in combination with other biomaterials, in biomedical research areas. The Section 1 gives a general overview of chitosan, and its structural, physical, and chemical properties detailing the extraction, properties, and various methods for its modification and the implications in the desired biomedical applications. The Section 2 focuses on the current trends in chitosan applications in each biomedical domain. The Section 13 delves into the limitations and future potentialities of chitosan as a versatile biopolymer.

## 2. Properties of Chitosan

### 2.1. Physical Properties

Hydrophilic polymeric scaffolds derived from chitosan have a three-dimensional cross-linked structure. Due to their physicochemical and biochemical properties, chitosan hydrogels are manufactured via chemical or physical cross-linking amongst the polymer backbone. They are used in therapeutic applications. Because of their capacity to regulate drug release using pH-responsive and temperature-responsive release techniques, as well as the networks that can carry active pharmaceutical compounds, chitosan hydrogels are helpful in drug delivery. Chitosan hydrogels are also a good alternative for wound healing due to their strong antibacterial properties, ability to provide humidity and heat to the wound, cytocompatibility, etc. Additionally, the swelling ratio, porosity, and mechanical behavior of these hydrogels make them an excellent choice for use as a tissue regeneration scaffold [20].

#### 2.1.1. Solubility

Chitosan is perhaps the most significant chitin derivative and the inclusion of numerous functional units on the polysaccharide backbone of this material, such as the hydroxyl and amine groups, allows for the creation of molecularly imprinted polymers and morphological changes [21]. Each D-glucosamine monomer has a free amino position, which could also become positively charged and provide essential features to chitosan, including solubility and antibacterial activity [22,23]. These moieties form excellent chelating ligands that can bind to several metal ions and electrostatically precipitate the dye anions. Furthermore, these amino units may be protonated, resulting in chitosan’s solubility in a dilute acid medium [24,25].

Chitosan is water-insoluble and insoluble in most liquid organic media; nevertheless, it is soluble in various aqueous acidic media below its pKa (pH = 6.5), including lactic acids, acetic acid, formic acid, and citric acid, as well as 10-camphor sulfonic acid, p-toluene sulfonic acid, and dimethyl sulfoxide. Carboxymethylation, quaternization, and phosphorylation of chitosan are structural modifications that enhance the polymer’s solubility in various solvent systems at atmospheric temperatures [26].

Chitosan’s solubility may be improved by lowering its molecular mass. The concentration of N-acetylglucosamine chains in chitosan is affected by molecular weight, which has intramolecular and intermolecular effects, leading to diverse chitosan morphologies. Nevertheless, regulating the deacetylation improves solubility at the expense of yield [27]. Breaking down the chitosan crystalline structure expands the spectrum of chitosan solubility. The researchers looked at both the physical as well as chemical ways of increasing chitosan solubility. Re-acetylation enhanced chitosan’s solubility until pH = 7.4 in their chemical method. The physical strategy included the utilization of admixtures with the ability to disturb the intra- and intermolecular hydrogen bond interactions, including urea and guanidine hydrochloride [28]. Chitosan’s solubility can be dramatically increased by adding smaller reactive groups to its structure, including alkyl (hydroxypropyl chitosan or carboxymethyl groups) [29]. Implementing a series of low MW chitosan compounds, a quick and efficient method of manufacturing solubilized chitosan (half N-acetylated chitosan) was devised [30].

#### 2.1.2. Viscosity

According to Kramer [η]_Kra_ and Huggins [η]_Hug_, the intrinsic viscosity of chitosan in a buffered aqueous solution (0.3/0.3 M CH_3_COOH/CH_3_COONa) was 646 and 637, respectively [12]. Kasaai et al. studied the relationship between intrinsic viscosity and the molecular weight of shrimp shell-derived chitosan in 0.25 M acetic acid/0.25 M sodium acetate solution. The Mark–Houwink–Sakurada equation (MHS) was suggested for chitosan between molecular weight ranges of 35–2220 kDa. The exponent α in the MHS indicated that chitosan behaved as a flexible chain in the solvent composition. The α and K are inversely proportional and depend upon the degree of deacetylation, pH, and ionic strength of the solvents [31].

### 2.2. Chemical Properties

The degradation products of chitosan called chito-oligosaccharides are water-soluble, have no cytotoxicity to organisms, are easily absorbable through the intestines, and are eliminated through the kidneys. Chito-oligosaccharides (COS) offer a plethora of biological properties, such as cholesterol-reducing activity, anticancer activity, and immunomodulatory activity [32].

### 2.3. Biodegradability

Many studies have looked at the biodegradability of chitosan. Under certain conditions, lysozyme [33], proteases [34], and porcine pancreatic enzymes were discovered to be capable of degrading chitosan [35]. The *Aspergillus niger* pectinase isozyme was further demonstrated to degrade chitosan at low pH, leading to lower MW chitosan [36,37]. Connell et al. employed human feces to show that chitosan-based films, glutaraldehyde polymerized films, and tripolyphosphate crosslinked films degraded significantly [38]. According to Brenner et al., many of the enzymatic catalysts appear to have an effect against chitosan, particularly in vitro; however, variants could result in indigestible compounds. To be therapeutically effective, these chemical particles must be sufficiently tiny to be eliminated by the kidneys (< 42 Å for neutral compounds). The bio-distribution is controlled by the mode of delivery, dose form, and chitosan properties, i.e., due to the physical qualities, the film/dense matrices will exist at the location of the application. The deterioration of the matrix can be controlled by changing the amine units’ crosslinking. Particle size (less than 100 nm) can affect intravenous diffusion, while molecular mass can dictate particle lifespan [39].

### 2.4. Toxicity

Chitosans’ toxicity was studied in several ways, such as in guinea pigs, frogs, human nasal palate tissues, and the nasal mucosa of rats. Toxicity was minimal in each experiment [40,41,42]. Ribeiro et al. investigated the in vitro cytotoxic effects of chitosan-based hydrogels using rat-skin dermal fibroblasts. The hydrogel, as well as its breakdown by-products, were shown to be non-cytotoxic in a cell viability assay. The total absence of a reactive or granuloma-forming inflammatory reaction in skin infections treated with chitosan biomaterials and no pathological irregularities in the organs corroborated the hydrogel’s systemic and local histocompatibility [43].

The intravenous injection of chitosan (4.5 mg/kg/day for 11 days) to rabbits resulted in no aberrant alterations, according to in vivo chronic toxicological tests. Data are generally scarce on chitosan’s toxic effects from human research [44]. According to Gades et al., the human participants who consumed more than 4.5 g of chitosan per day did not experience any harmful consequences. Even greater oral doses of up to 6.75 g were found to be safe [45]. Brief human testing lasting up to 12 weeks has revealed no clinically substantial effects, such as no signs of an allergic reaction [46]. The safety of chitosan mouthwash was determined using the Ames, MTT, and V79 chromosomal abnormality studies. The chitosan-based mouthwash was less toxic and had more decisive antibacterial action than the conventional mouthwash. Moreover, unlike commercialized mouthwash, this was found to suppress two pathogenicity indicators (streptococcus and enterococcus) while causing no significant changes in the survival of the typical oral microbiota [47].

Ravindranathan et al. examined pure, mild endotoxin chitosan and found that the viscosity/molecular weight and deacetylation degree within the limits of 20–600 CP and 80–97 percent, correspondingly, have not affected the immunogenicity of chitosan. Endotoxin exposure was expected to have a significant impact on immunogenicity. Only endotoxin concentration, deacetylation degree, or viscosity impacted on the chitosan-induced immune reactions, according to their findings. Their findings also showed that lower endotoxin chitosan (0.01 EU/mg) with viscosities of 20–600 cP and deacetylation levels of 80–97% is largely innocuous. This work emphasized the importance of more thorough identification and purification of chitosan in laboratory development before being employed in clinical trials [48].

According to Baldrick, chitosan could be employed as a non-parenteral and non-blood constitutive medicinal excipient. The appropriate use of chitosan as an injectable excipient is not apparent depending on the existing data. The material’s hemostatic physiological nature allows it to be used as a medical device to stop hemorrhaging, and investigations have shown local findings, such as blood clotting, thrombosis development, and platelet adhesion [49]. Despite some cytotoxicity observations in vitro, there are still instances of non-toxic chitosan used in medicines, such as to halt blood loss [50,51,52].

## 3. Methods of Preparation

Chitosan is naturally derived from the polysaccharide chitin, which is the second most abundant bio polysaccharide, generally seen in the shells of lobsters, shrimps, crabs, tortoises, and even insects [53]. Chitosan is produced by the physical or chemical deacetylation of chitin, and even though an established definition of chitosan does not exist, it is generally accepted that 70% deacetylated chitin is chitosan [54]. However, in commercial applications, a degree of deacetylation (DD) of 70–90% or even higher may be desirable by undergoing subsequent deacetylation steps [55]. However, this process may also lead to polymer degradation and increased chances of reacetylation. Hence, chitosan’s molecular weight is generally dependent on its DD. The lower the DD, the higher the MW, which imparts more significant chemical and mechanical stability but also decreases its solubility in most solvents in regular use. This deacetylation reaction is ideally carried out in a nitrogen-rich environment or by adding it to a mixture of NaOH and sodium borohydride to avoid any side reactions from taking place. In this way, the chitosan produced has an average molecular weight of 1.2 × 10^5^ gmol^−1^ [55].

Throughout the past decades, scientists have explored and implemented several ways of extracting chitosan from the shells of various crustaceans, insects, and fungi [56]. Chitosan biopolymers can also be produced from *Labeo rohita’s* discarded scales [57]. To produce chitosan from chitin, two techniques with differing degrees of acetylation had proved to be widely employed. The first is the heterogeneous deacetylation of dry chitin, while the second is the uniform deacetylation of pre-swollen chitin in an aqueous solution in a vacuum [58]. The deacetylation procedure requires strong alkali treatments and extended operating durations in both of the circumstances. The production time is determined by whether the circumstances are heterogeneous or homogeneous and can range from 1 to over 80 h. Alternative manufacturing procedures have been devised to lessen the relatively long processing time and significant volume of alkali. Employing thiophenol in DMSO for sequential alkali processes [59]; thermo-mechanical methods employing a cascade reactor maintained at a low alkali percentage [60]; flash procedures under saturated steam [61]; microwave dielectric heating [62]; and periodic water washing [63,64] are other instances of alternative manufacturing procedures. Previous studies have revealed various sophisticated chitosan recovery strategies involving high-energy bombardment. Microwave radiation is a common alternative energy source that can transmit power directly and fast into the substrates, enhancing reaction efficiency [65]. Furthermore, microwave treatment can minimize the number of chemical compounds employed in the chitosan extraction method; nevertheless, the DD of chitosan obtained is not convincing. Rashid et al. described a –irradiation methodology for making chitosan from prawn shells that substantially increased chitin’s DD while using a low alkali quantity [56].

## 4. Extraction

### 4.1. Deproteinization

Desiccated crustacean shells will be initially washed with an alkaline solution (e.g., NaOH, KOH, etc.) to eliminate the proteins. The alkali-insoluble portion is then separated by centrifugation, accompanied by repeated washings with distilled water till the pH reaches neutrality [53].

### 4.2. Desulfurization

Next, the mineral contaminants are removed from deproteinized shells using a dilute mineral acid (e.g., HCl). The acid-insoluble portion is then separated using centrifugation. The acid is rinsed out of the isolated fraction using distilled water. The chitin, which is somewhat pink in color, evaporates to dryness overnight [53].

### 4.3. Decolorization

The chitin is decolorized by reacting with an oxidizing agent such as potassium permanganate, hydrogen peroxide, or other oxidizing agents, then washed with an oxalic acid solution. Purified chitin is the name given to the end product [53].

### 4.4. Deacetylation

The decolorized chitin would then be treated with highly alkaline solutions over many hours to deacetylate it and transform it into chitosan. Centrifugation separates the alkaline fraction of the combination, and excessive alkali is discharged with a rinse of distilled water till the pH becomes neutral. The chitosan portion recovered is then dried and kept at ambient temperature. Raw chitosan is diluted with aqueous 2 percent (*w*/*v*) acetic acid to get the optimum product. The insoluble substance is then filtered, yielding a precise supernatant mixture neutralized with NaOH solution, yielding a pure chitosan specimen as a colorless precipitate. The chitosan appears in flakes that range in color from white to yellow and can be made into beads or powder form. To create medicinal and pharmaceutical-grade chitosan, more purification may be necessary [53].

## 5. Modifications of Chitosan

In recent years, chitosan has been increasingly utilized in the pharmaceutical and biomedical fields because of its many advantages, such as low immunoreactions, good biocompatibility, easy biological degradability, excellent mucoadhesion, and its natural abundance [66]. The versatile biological and physicochemical characteristics of chitosan are possible because of the availability of different functional groups. These groups can be functionalized by a variety of processes, such as the Schiff’s base chemical reaction of aldehydes or ketones with the -NH_2_ functional group, carboxymethylation, acetylation, quaternization, chelation with metals, alkylation, and sulfonation, etc. [2,67].

In its native form, chitosan has three terminal functional groups at distinct places: the C6-OH group, C3-OH group, and the C2-NH2 group, of which the C2-NH2 and the C6-OH groups could be easily modified, but the modification at the C3-OH site is not favorable because of the higher steric hindrance [68]. Naturally, the amino groups are the most common modifications made because of the feasible reactivity of the C2-NH2 group, making the grafting reactions much simpler. These amino groups are slightly more reactive with nucleophiles; nonetheless, both the hydroxyl and amino groups can readily react in electrophilic reactions with reagents such as acyl chlorides, acids, and alkanes, which can lead to the functionalization of the OH and NH2 groups in a non-selective manner [68]. Several reactive functional groups allow chitosan to interact with proteins and gain a cationic character, enhancing the adherence and differentiation of cells. Apart from this, the poor aqueous solubility of chitosan is an issue, which limits chitosan’s biomedical utility, especially in physiological conditions, wherein it is poorly soluble and thus becomes a poor absorption promoter [69]. In addition, chitosan’s efficiency of transfection is relatively low, and many beneficial functionalities are absent in chitosan, severely limiting its applications. Hence, it becomes imperative to modify the chemical characteristics of chitosan or to add some desirable functionalities, which will increase the water solubility for various therapeutic applications and offer a potential resource for endorsing novel biochemical actions while enhancing its material properties [70]. Astonishingly, it has been seen that modifying the structural features alone did not cause any significant change in chitosan’s basic properties, but it does give them new properties. Chitosan has a unique structure that allows it to undergo various reactions, such as halogenation, oxidation, reduction, cross-linking, complexation, phosphorylation, and acylation, which impart new properties to these derivatives [68]. Thus, chitosan, along with its derivatives, has been famous for its tunable biological and chemical characteristics. Compared to native chitosan, their functionalized analogs have quicker gel-formation properties, greater aqueous solubility, and the capability of forming self-assembling nanostructures. Additionally, the design of hydrophobic equivalents with amphiphilic properties and chemical groups with a wide range of medicinal and active compounds; improved DNA complexing characteristics; and improved bioactivity [71].

Quaternized chitosan-modified black phosphorus nanosheets (BP-QCS) were prepared by Zhang et al. by electrostatic adsorption, wherein BP-QCS had more chemical stability and dispersiveness than plain BP in an aqueous medium. BP-QCS also had good biocompatibility, and photothermal/pharmacologic combination antibacterial action under NIR radiation (98% *S. aureus* inactivation in vivo) [72].

## 6. Antimicrobial Properties of Chitosan

Researchers have been quite interested in the antibacterial properties of chitosan as well as its analogs. Chitosan was shown to have antimicrobial inhibitory activity against bacteria, filamentous fungus, and yeast strains. Chitosan has also been identified as an antibacterial agent, although its ability to work in this manner is still unknown, as various distinct processes have been ascribed to its character. According to one theory, chitosan stimulates the migration of Ca++ from anionic locations of the membranes when subjected to the bacterial cell walls, leading to cell damage. It additionally has antiplaque efficacy against *Porphyromonomas gingivalis, Prevotella intermedia*, and *Actinobacillus actinomycetemcomitans*, among other oral infections [73,74,75].

Chitosan has a broad spectrum of action and high mortality towards gram-positive and gram-negative microorganisms, while being relatively nontoxic to mammals. Chitosan’s bactericidal properties are said to be based on its molecular size. No et al. tested the antimicrobial property of six chitosan oligomers with wildly differing molecular masses against gram-positive and gram-negative bacteria to verify this theory. They discovered that chitosan significantly slowed the development of most bacteria examined, albeit the inhibitory effects varied depending on molecular size and strain. For gram-positive bacteria, chitosan had a more significant bactericidal impact than gram-negative bacteria. The explanation for this disparity is unknown; however, Y. J. Jeon et al. discovered that oligosaccharides and chitosan had stronger inhibitory activity against gram-positive microorganisms than gram-negative ones. As a result, it is understandable that chitosan has a strong inhibitory effect on gram-positive lactobacilli [28,76].

## 7. Applications in Drug Delivery

In recent years, nanocarrier-based drug delivery systems have become increasingly popular for the administration of active compounds to the desired site of action [77,78,79,80,81,82,83,84]. Numerous pieces of research have been conducted to determine chitosan’s effectiveness as an orally administered vehicle [75,85]. The use of such medication carriers reduces the risks of systemic delivery [86]. Chitosan-based composite materials can be used to create reliable local drug carriers with the necessary mechanical behavior, retention time, and extended-release pattern [87]. Chitosan microspheres were designed to actively deliver therapeutics in disease areas [88,89]. Chitosan is non-toxic when taken orally and approved as a food additive by the Food and Drug Administration (FDA). It has also been investigated as a drug carrier for various macromolecules, including DNA, siRNA, growth regulators, and a variety of therapeutics [90,91].

Over the years, chitosan has been utilized in nanotechnology-based formulations, such as nanoparticles for drug, protein, and gene delivery through various routes of administration such as oral, topical, and parenteral routes [11]. To enhance the stability of chitosan nanoparticles (NPs), Saeed et al. used polyphosphoric acid or hexametaphosphate for concurrent ionotropic/covalent crosslinking with chitosan NPs. The resultant NPs showed considerable stability under CaCl_2_, 10% fetal bovine serum, and harsh pH conditions [92]. Similarly, Yu et al. synthesized octenyl succinic anhydride-modified chitosan nanoparticles to improve the anti-inflammatory and antioxidant properties of two model drugs: quercetin and curcumin. The as-prepared NPs exhibited pH-dependent release with faster drug release achieved at around pH 6.0 [93]. Thus, the versatile physicochemical properties and the tunable nature of chitosan renders it as a great candidate for nanotherapeutic drug delivery.

In a study by Barbosa et al. quercetin was delivered using novel polymeric nanoparticles derived from fucoidan and chitosan. Fucoidan/chitosan (F/C) nanoparticles, having three distinct weight fractions (1/1, 3/1, and 5/1), were produced by Barbosa et al. using the polyelectrolyte self-assembly approach. On increasing the mass ratio of fucoidan to chitosan inside the nanoparticles, the amount of quercetin in the fucoidan/chitosan nanoparticles ranged from 110 ± 3 to 335 ± 4 mgmL-1. With the size of the nanoparticles in the 300–400 nm region and membrane potential of more than +30 mV for the 1F/1C proportion nanoparticles and about 30 mV for the 3F/1C and 5F/1C ratio nanoparticles, the physicochemical characteristics of stable nanoparticles were developed. As the pH rose from 2.5 to 7.4, the 1F/1C ratio nanoparticles grew larger and much more unstable, but the 3F/1C and 5F/1C nanoparticles remained unchanged. This showed that the latter nanoparticles remained stable throughout the digestive tract. Within simulated gastrointestinal conditions (particularly for the 3F/1C and 5F/1C combinations), the quercetin-loaded fucoidan/chitosan nanoparticles demonstrated significant antioxidant potential and controlled delivery, limiting quercetin deterioration and improving its oral absorption [94].

**Figure 1:**
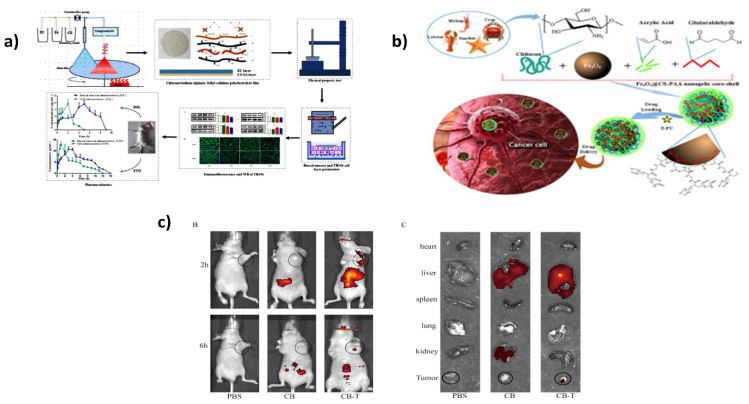
(**a**) Interfacial reaction solvent-drying method for EC-SA-CS polyelectrolyte film via self-made equipment [95]; (**b**) Preparation and characterization of chitosan-based magnetic nanohydrogels for 5-fluorouracil drug administration and a kinetic assessment. Reproduced with permission from [97], copyright Elsevier 2022; (**c**) (B) Tumor-infected animals were given Cy5.5-labeled nanoparticles intravenously. The biodistribution of various compositions in vivo at 2 and 6 h, (C) ex vivo imaging of main organs and tumors. Reproduced with permission from [103], copyright Elsevier 2021.

The fabrication of a film containing chitosan, sodium alginate (SA), and ethyl cellulose (EC) for buccal mucosa delivery was described in a study by Wang et al. (as depicted in Figure 1a). Utilizing self-made equipment, an interfacial reactive solvent-drying process was used to create a film of CS-SA unilateral releasing drug-loaded water-repellent layer EC. When matched to CS-EC and SA-EC films, the CS-SA-EC film had excellent tensile qualities. FT-IR acknowledged the formation of the amide linkage. DSC revealed that the active ingredient was distributed in an amorphous state inside the carrier system. The model compounds from the CS-SA-EC films had superior release qualities, according to the in vitro drug release study. All of the combinations of the drug release pathways are best described by the Ritger–Peppas theory. The films’ permeation properties were tested using the TR146 cells culture and rabbit buccal mucosae through immunofluorescence and Western blotting. The prototype drugs’ aggregate permeation levels were dramatically boosted. The film suppressed the translation of ZO-1 protein, and the ZO-1 protein expression pattern agreed with the results of the in vitro penetration tests. In rat models, the bioavailability of the drug-loaded films was assessed and compared to oral delivery. Compared to oral delivery, the relative absorption of the model medicines was 246.00 percent (Zolmitriptan) and 142.12 percent (Etodolac). The findings of this investigation show that the CS-SA-EC carrier has the capacity to transport drugs to the mucosal layer [95].

Chemotherapy is presently employed for most cancer therapies, but one of the substantial drawbacks of this approach is that it harms the body’s normal tissues [96]. As a result, a few of the globe’s most significant problems are developing new mechanisms for the smart and targeted delivery of these medications in tumor tissue. As a result, substantial money is now being spent on developing innovative drug delivery systems (DDS) featuring targeted delivery (as shown in Figure 1b). In a study by Kheiri et al., glutaraldehyde was employed to produce chitosan-polyacrylic acid-encapsulated Fe3O4 magnetic nanogelic core-shell (Fe3O4@CS-PAA) for carrying anticancer 5-fluorouracil (5-FU) medication. Then, the drug carrier assays were performed in an in vitro setting that mimicked a physiologic microenvironment and tumor tissue parameters. The Fe3O4@CS-PAA improved the rates of 5-FU release from nanogelic core-shell under tumor tissue settings (pH 4.5) compared to physiological fluids (pH 7.4). A variety of models were also employed to examine the drug release process. The process of 5-FU release from Fe3O4@CS-PAA was governed by Fickian diffusion [97].

Classic chemotherapy medicines for lung carcinoma have several drawbacks, including harsh side-effects, unpredictable drug release, low absorption, and resistant strains [98,99,100,101,102]. To overcome the constraints of unloaded drugs and enhance treatment outcomes, Zhu et al. created novel T7 peptide-based nanoparticles (T7-CMCS-BAPE, CBT) premised on carboxymethyl chitosan (CMCS), which were also likely to bind to the transferrin receptor (TfR) presented on lung carcinoma cells and accurately control the drug release depending on the pH and reactive oxygen species (ROS) levels. The drug-load content of docetaxel (DTX) and curcumin (CUR) was nearly 7.82 percent and 6.48 percent, respectively. Substantial biosafety was achieved even at concentrations as high as 500 g/mL. Notably, the T7-CMCS-BAPE-DTX/CUR (CBT-DC) combinations outperformed DTX solo therapy and other nanostructures loaded with DTX and CUR alone in vitro and in vivo studies. Additionally, they discovered that CBT-DC could improve the immunosuppressive surroundings, promoting tumor inhibitory activity (as illustrated in Figure 1c). These findings help set the groundwork for multimodal anticancer therapy [103].

p-mercaptobenzoic acid-embedded N, N, N-trimethyl chitosan nanoparticles (MT NPs) were effectively synthesized by Zhang et al. to carry anticancer medicines, genes, and immunological agents in a homogeneous nanoparticulate platform. Paclitaxel (PTX) was entrapped in the hydrophobic cavity of the MT NPs, while the hydrophilic exterior of the MT NPs had been loaded with survivin shRNA-expressing plasmids (iSur-pDNA) and recombinant human interleukin-2 (rhIL-2). The large quantity of glutathione induced a fast release of PTX due to the redox sensitivity of MT NPs. The MT/PTX/pDNA/rhIL-2 NPs were provided with higher anticancer therapeutic efficacy and enhanced tumor-induced immune responses resulting from the combined effects of PTX (1.5 mg/kg), iSur-pDNA (1.875 mg/kg), and rhIL-2 (6 × 10^5^ IU/kg) at low dosages. The co-delivery of PTX, iSur-pDNA, and rhIL-2 by amphipathic chitosan-derived NPs with redox sensitivity could be a potential mechanism in the therapy of malignancies [104].

In addition to the above, several other studies that were carried out to facilitate drug delivery by chitosan-based carriers include aluminum-modified mesoporous silica nanoparticles (H/Al-MSN)/curcumin/chitosan/mesalamine [105]; chitosan/polyvinyl pyrrolidone (PVP)/5-Fluorouracil [106]; quaternized chitosan/thiolated carboxymethyl chitosan [107]; chitosan/aptamer/mesoporous silica nanoparticles/doxorubicin [108]; norborene functionalized chitosan (CsNb)/polyacrylic acid (PAA)/5-Amino salicylic acid [109]; chitosan/pectin/5-Fluorouracil [110]; chitosan/dopamine/inulin aldehyde/indomethacin [111]; chitosan/magnetic alginate/amoxicillin [112]; chitosan/PVP/α-Fe2O3/doxorubicin [113]; and chitosan/mesoporous silica/methotrexate [114].

## 8. Applications in Gene Delivery

In the recent decade, the applications of RNA-interfering agents in gene therapy have been developed to exponential levels. Nevertheless, the tumor-targeting potential of these small interfering RNAs (siRNAs) is still a bottleneck [115,116,117]. Cancer progression involves various stages; caspases-linked anti-apoptotic factors inhibit apoptotic protein expression. One such gene, the survivin gene, is implicated in several physiological processes, such as regulating the cell cycle, cellular protection, and apoptosis suppression; these processes ensure that the cancerous cells survive [118,119]. Chitosan and polyethylene glycol (PEG) are known to aid the synthesis of cationic oligonucleotide nanoparticles [120,121]. Polyethyleneimine (PEI), an efficient gene carrier owing to its proton-sponge effect, functions as a buffer surrounding the endosome and delivers substances into the cytoplasmic space [122]. PEG helps reduce PEI toxicity, facilitates the formation of stable colloids, and prevents the deposition of nanoparticles [123]. The nanoparticles are coated by the chitosan, thus stabilizing them and preventing agglomeration. Hence, Arami et al. fabricated Fe3O4-PEG-LAC-chitosan-PEI nanoparticulate carriers, which were sufficiently cationic to react with siRNA. In vitro, they transferred the survivin siRNA to human breast cancer cells (MCF-7) and human chronic myelogenous leukemia cells (K562) using the nanoparticulate carrier. They found that the Fe3O4-PEG-LAC-chitosan-PEI nanoparticles combined sufficiently with siRNA, their sub-nanomolar size made them suitable gene carriers, and the survivin siRNA therapy was biocompatible and non-toxic to healthy cells [124].

**Figure 2:**
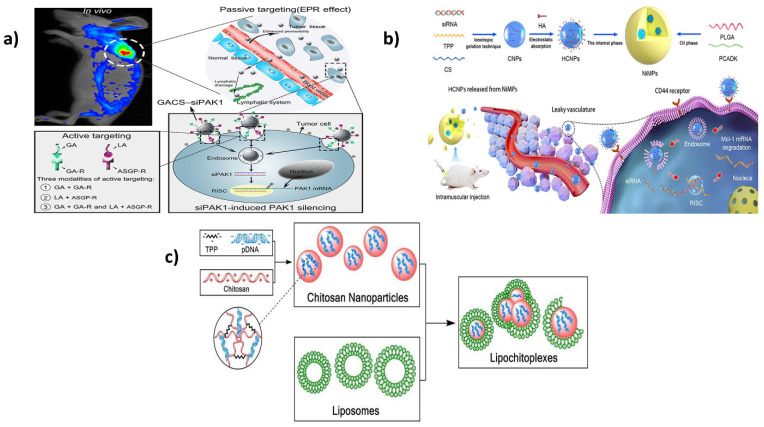
(**a**) Delivery of siRNA via Dual-Targeting Nanoparticle-based Gene Therapy for Hepatocellular Carcinoma [128]; (**b**) A systematic approach for rheumatoid arthritis treatment using PLGA/PCADK hybrid microspheres encapsulating hyaluronic acid–chitosan siRNA nanoparticles. Reproduced with permission from [129], copyright Elsevier 2021; (**c**) Improved gene delivery with lipid-enveloped chitosan-DNA nanoparticles. Reproduced with permission from [130], copyright Elsevier 2018.

Chitosan has shown potential for protecting siRNA from plasma denaturation and delivery into cancer cells by promoting the deposition of antineoplastic agents and biomolecules in the solid tumor tissues via the enhanced permeability retention (EPR) pathway [125]. Even though chitosan can be endocytosed by a ligand-receptor-mediated mechanism [126], the mono-ligand uptake of NPs is limited due to the saturation of membrane receptors [127]. RNA interference as a gene delivery method is limited by its low ability for targeted therapy and cellular absorption of small interfering RNA (siRNA). Hence, Zheng et al. fabricated chitosan-based dual-ligand nanoparticles (NPs) (GCGA) loaded with siPAK1 (GCGA-siPAK1), as shown in Figure 2a, whereby the targeting activity was influenced by the ligand molecules of glycyrrhetinic acid (GA) and galactose of lactobionic acid (LA). The NP targetability and siPAK1 cellular uptake were enhanced in the hepatocellular carcinoma by the GCGA-siRNA system [128].

In another study, Zhao et al. utilized the cationic chitosan’s ability as a siRNA vector modified using a targeting molecule such as hyaluronic acid (HA), which has an affinity toward CD44 receptors expressed on activated macrophages. They developed a sustained-release composite MP system using poly (cyclohexane-1, 4-diylacetone dimethylene ketal) (PCADK)-loaded HCNPs and PLGA-based siRNA therapy for rheumatoid arthritis (as depicted in Figure 2b). The HCNPs were prepared by the ionotropic gelation method as a barrier for nuclease-mediated siRNA degradation and were then loaded as the aqueous phase into 20% PCADK and PLGA MPs, forming an NP-in-MP composite system (NiMPs) without disturbing the pH microenvironment inside the MPs. However, the introduction of HCNPs made the repulsion into attraction because of the surface positive charge on chitosan and changed the siRNA distribution from the periphery into a uniform distribution. In vitro release of siRNA was sustained release of 70% in 15 days. In vivo experiments on rat models revealed that siRNA had a relatively similar concentration in blood for up to 8 days and the pharmacodynamic effects were the same as HCNPs [129].

Chitosan offers the benefit of a biodegradable and highly biocompatible biopolymer when used as a polycationic non-viral vector for gene transfer. Nevertheless, owing to its poor ability to successfully transfect under biological settings, it is of little value as a genetic delivery device without arduous chemical alterations to its composition. To solve this issue, Baghdan et al. created lipochitoplexes, which are liposome-encapsulated chitosan nanoparticles (LCPs), as shown in Figure 2c. The ionotropic gelation process was used to develop chitosan nanoparticles (CsNPs). A polyanionic tripolyphosphate was used for cross-linking the low molecular weight chitosan with a high DD, resulting in the effective trapping of plasmid DNA (pDNA) within the nanoparticles. The chitosan nanoparticles were incubated with anionic liposomes (DPPC/Cholesterol) to make LCPs. In physiological environments, the LCPs provided excellent pDNA protection, lower cytotoxicity, and a twofold improvement in transfection efficiency. In the chorioallantoic membrane model (CAM), the efficacy of the delivery vector was also demonstrated in vivo. The LCPs could transfect the CAM without causing any damage to the nearby vascular capillaries. This unique biocompatible hybrid framework, free of chemical alterations, organic solvents, or harsh manufacturing processes, was deemed an ideal gene delivery mechanism for in vivo studies, revealing new information about non-viral therapeutics [130].

Other such studies related to gene delivery using chitosan are PEGylated chitosan/CRISPR-Cas9 dry powder [131]; carbonized chitosan/zeolite imidazolate nanoparticles/luciferase-expressing plasmid (Pgl3)/splice correction oligonucleotides (SCO) [132]; methyl methacrylate-based chitosan/DNA [133]; cell-penetrating peptide-loaded chitosan-based iron oxide nanoparticles/plasmid pGL3/small interfering RNA/splice correction oligonucleotides [134]; thiolated trimethyl aminobenzyl chitosan/methylated 4-N,N-dimethyl aminobenzyl N,O carboxymethyl chitosan/thiolated trimethyl chitosan/plasmid DNA [135]; alkylamine-modified chitosan/p53 [136]; chitosan nanoparticles/polyethylene glycol/poly lactic acid/nerve growth factor/acteoside/plasmid DNA [137]; chitosan/5-Amino-tetrazole(3-Chloropropyl)trimethoxysilane/Fe3O4 [138]; chitosan/starch polyplexes/plasmid DNA [139]; and chitosan/gelatin/oxidized sucrose/timolol maleate [140].

## 9. Applications in Protein Delivery

**Figure 3:**
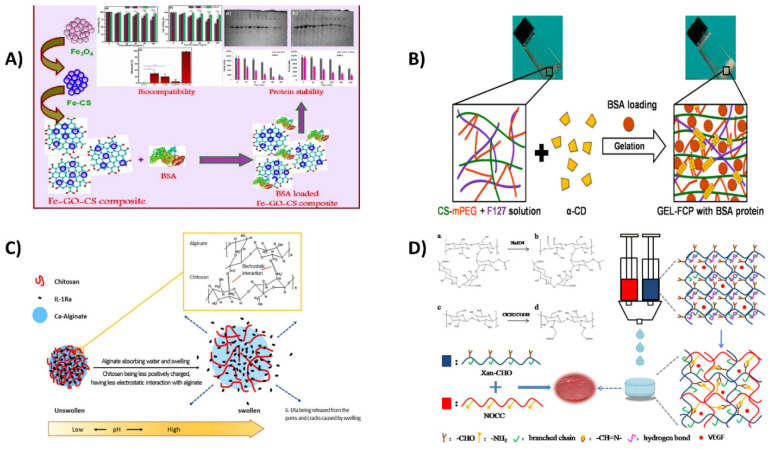
(**A**) A graphene oxide–chitosan combination with magnetic nanoparticles as a protein-delivering vehicle. Reproduced with permission from [141], copyright Elsevier 2021. (**B**) Fabrication of Chitosan–PEG conjugates and its Supramolecular Hydrogels for Protein Delivery Using Sulfur (VI) Fluoride Exchange (SuFEx) [144] (**C**) In situ application of the protein interleukin-1 receptor antagonist (IL-1Ra) for the therapy of dextran sulfate sodium (DSS)-induced colitis in a murine model using alginate/chitosan microcapsules. Reproduced with permission from [145], copyright Elsevier 2019. (**D**) For localized drug delivery, a unique in situ forming hydrogel consisting of xanthan and chitosan re-gelifying in liquids. Reproduced with permission from [146], copyright Elsevier 2018.

Protein therapies have gained much traction in the medical business to fight diseases such as cancer, digestive problems, and autoimmune conditions. Protein distribution in vitro and in vivo, on the other hand, is hampered by protein degradation and a shorter lifetime. As a carrier system for protein, Rebekah et al. developed magnetic nanoparticles coated with graphene oxide chitosan hybrid (Fe-GO-CS), as shown in Figure 3A. To investigate the integrity and function of the produced nanocarrier, bovine serum albumin (BSA) was used as a protein. After 30 min and 3 h of exposure to Fe-GO-BSA and Fe-GO-CS-BSA solutions in trypsin, the SDS-PAGE examination revealed no significant changes. When relative to the Fe-GO composites, the Fe-GO-CS has a better drug load and release pattern, and the carrier system preserves the peptide from proteolytic action. As a result, the Fe-GO-CS composite provides a superior nanocarrier that can be used in therapeutic settings [141].

Salivary proteins, such as histatins (HTNs), have been shown to have important physiological activities in dental homeostasis and avoiding periodontal disease. HTNs, on the other hand, are vulnerable to the mouth environment’s considerable proteolytic activity. To preserve peptides from hydrolytic enzymes at a normal salivary pH, pH-sensitive chitosan nanoparticles (CNs) have been proposed as possible carriers in a study by Zhu et al. The optimized formulations had a batch-to-batch consistency of 144 ± 6 nm, a polydispersity value of 0.15 ± 0.04, and a zeta potential of 18 ± 4 mV at a maximum pH of 6.3. Cationic polyacrylamide gel electrophoresis was used to examine HTN3 entrapment and release characteristics. HTN3 was successfully entrapped by the CNs, which were swollen exclusively at lower pH to aid HTN3 release. In diluted whole saliva, the stability of HTN3 against proteolysis was examined. Compared to unbound HTN3, HTN3 enclosed in CNs showed a longer lifetime. Biofilm density and microbial vitality were likewise lowered by CNs both in the presence and absence of HTN3. The study’s findings showed that CNs are suitable as prospective protein carriers for oral purposes, particularly in the case of the difficulties that emerge under acidic circumstances [142].

Because of their versatility, supramolecular hydrogels are considered attractive drug vehicles for tissue regeneration [143]. Chitosan hydrogels lacking chemical cross-linkers are low in cytotoxicity and offer a high distribution potential, but they have poor mechanical qualities for injectable hydrogels. Jang et al. used click chemistry to create novel chitosan analogs for constructing supramolecular hydrogels with greater structural rigidity under mild circumstances (as depicted in Figure 3B). A sulfur–fluoride exchange process was used to synthesize the chitosan derivatives effectively, and the resulting chitosan-mPEG/Pluronic-F127 (CS-mPEG/F127) bonded with -cyclodextrin (-CD) to produce a supramolecular hydrogel through a host–guest interaction. The proportion of chitosan-mPEG and F127 could influence gelation kinetics, hydrogel characteristics, and bovine serum albumin (BSA) release. Thus, supramolecular hydrogels represent potential long-term tissue regeneration protein carriers [144].

The effective therapy of irritable bowel syndrome can benefit from the targeted administration of bioactive molecules such as proteins to the colonic region. Hence, Cao et al. used a single-step electro-spraying approach to make alginate/chitosan microcapsules (Alg/Chi/IL-1Ra MC) encapsulating IL-1Ra, as shown in Figure 3C. The pH-stimulation of the microcapsule and the in vitro release pattern was evaluated as critical efficacy considerations. The therapeutic efficacy of the Alg/Chi/IL-1Ra microcapsules was assessed using the dextran sodium sulfate (DSS)-induced colitis murine model, and the findings revealed that the Alg/Chi microcapsules shielded IL-1Ra from the hostile conditions of the upper gastrointestinal region. This was due to the microcapsule’s pH-sensitive reaction, which enabled the targeted delivery of IL-1Ra in the colon. The Alg/Chi/IL-1Ra microcapsules reduced DSS-induced colitis in mice as measured by DAI, colonic length, colorectal tissue shape, histological injury ratings, and comparative protein concentrations (MPO, TNF-, and IL-1). This study indicated a viable method for oral protein administration, in situ colon release, and the potential use of IL-1Ra in treating autoimmunity and inflammatory illnesses [145].

Injectable hydrogels have long been a popular biomaterials subject. Unfortunately, due to tissue secretions, they are easily distributed during gelatinization in vivo, resulting in controlled-release drug delivery inability. To address this issue, Huang et al. described a new natural polymer-based injectable hydrogel made from aldehyde-modified xanthan (Xan-CHO) and carboxymethyl-modified chitosan (NOCC) that self-crosslinks, as illustrated in Figure 3D). The molar ratio of Xan-CHO and NOCC was adjusted to enhance the physical characteristics. Studies indicated that this composite material had self-healing, anti-enzymatic hydrolysis, biological compatibility, and biodegradability features. The release curve showed that BSA-FITC released in liquids was stable after 10 h. There was an association between this biopolymer and the hosts after integration with a vascular endothelial growth factor, which expedited the rebuilding of the abdomen wall in rats. As a result, from a carrier perspective, this injectable hydrogel could minimize drug eruption in a range of circumstances and serve as a tissue-building scaffold [146].

Other studies conducted for chitosan-based protein delivery were fluorinated chitosan-chlorine e6/catalase [147]; chitosan/tripolyphosphate nanoparticles/ selenomethionine [148]; chitosan/ multiwalled carbon nanotubes/arginine-glycine-aspartic acid (RGD)/ urokinase [149]; mannose-anchored quaternized chitosan/thiolated carboxymethyl chitosan [107]; glycol chitosan/telechelic difunctional poly(ethylene glycol)/doxorubicin/gemcitabine [150]; chitosan/mesoporous silica/oxidized sodium carboxymethyl cellulose/sodium hyaluronate/cytarabine/methotrexate [151]; chitosan/poly(N-isopropylacrylamide) (PNIPAAm)/methotrexate [152]; chitosan/graphene oxide/folic acid [153]; chitosan/Fe3O4 nanoparticles/oxaliplatin/irinotecan [154]; and chitosan/acrylic acid [155].

## 10. Vaccine Delivery

Traditionally, vaccines were considered for large-dose administration, with limited targetability and noticeable immunogenicity. Thus, they necessitated using immunity-modifying adjuvants to improve their specific immunity [156,157]. The vaccine technology has since come a long way, and now nano-vaccines have demonstrated some added advantages such as higher antigenic retention ability, convenient administration, better-targeted delivery, and enhanced bioavailability. Since chitosan has desirable properties, including its high biodegradability, non-toxicity, immunostimulatory activity, targeting potential, and provision for controlled vaccine release, it shows good potential for carrier material in nano-vaccines and their adjuvants. Chitosan-based materials also function as adjuvating agents in vaccines and impart both immunostimulatory and immunotherapy functions. In addition, chitosan-based nanogels demonstrate improved penetrability and controlled drug release features; additionally, they show antigen-storing ability, antigen presentation, and immunoregulation.

The nanoparticles containing chitosan having a net positive surface charge can improve the adhesiveness of antigenic substances to the nasal mucosa and thus augment its absorption, which is essential for intranasal vaccine administration. To this end, Gao et al. developed chitosan NPs via a gelation method and functionalized the chitosan NPs using mannose by a hybridization technique. Using bovine serum albumin (BSA) as the model antigen, they prepared an intranasal vaccine using an optimization-based design of experiments (DOE). It was found that the mannose-based chitosan NPs (Man-BSA-CS-NPs) demonstrated good modification ability and an average particle size distribution and surface charge of 156 nm and +33.5 mV, respectively. The release of BSA from the systems displayed no irreversible deterioration or agglomeration. Additionally, the fluorescence analysis revealed an excellent binding constant between BSA and CS, indicating that BSA had good stability. In vitro studies also show that the Man-BSA-CS-NPs were non-toxic and biocompatible. Moreover, the Man-modified BSA-FITC-CS-NPs promoted the endocytic uptake and internalization of BSA-FITC when tested in DC2.4 cells. More importantly, the Man-BSA-CS-NPs demonstrated significant immunogenic enhancement of the BSA-specific IgG titer and the highest BSA-specific IgA response in the nasal lavage samples of in vivo mice models. Thus, this study shows how modifying chitosan with sugars and proteins and loading into NPs could be useful in vaccine delivery [158].

Wei et al. evaluated the vaccine delivery potential of a hydrophilic ph-responsive phosphorylated CS (PCS) incorporating ovalbumin (OVA) antigen and tested it in vivo on mice models. PCS solution in the mice formed a dense gel-like OVA network, resulting in enhanced immunity. This was hypothesized to be because of the sustained and controlled release of OVA, giving lengthier immune protection. Moreover, hydrogels’ aqueous solubility enabled a convenient and hassle-free vaccine administration. Hence, because of the biocompatibility and non-toxicity of pH-responsive CS, hydrogels could be a potential platform for vaccine delivery [157]. However, their instability in solution and in vivo systems limits their overall applicability. Researchers suggest that modifying chitosan’s hydrophilic surface moieties, deacetylation, or reduction in its MW may improve its water solubility. Otherwise, chemical alterations to its structure could be attempted to prevent its in vivo degradation [157].

Zho et al. developed a thermoresponsive hydrogel comprising N-(2-hydroxypropyl)-3-trimethylammonium chitosan chloride (HTCC) and α-β-glycerophosphate (α-β-GP) as the vaccine carrier system for the *C. psittaci* antigen against avian influenza. The intranasal mucosal route of administration of vaccine gave the highest immune response in chickens [159].

Since the positively charged chitosan can easily interact with cellular membranes, contributing to its high biodegradability and formability, Zhang et al. prepared a chitosan-based PLGA nanoparticle vaccine encapsulating the recombinant protein OmpAVac (Vo) (VoNPs) against the *Escherichia coli* K1 caused meningitis in mice. The freeze-dried VoNPs were immunomodulatory in mice even after 180 days of storage [160].

Table 1 focuses on recent investigations on chitosan formulations for delivering active pharmaceutical compounds.

## 11. Tissue Engineering

A recently blooming field of research for regenerating injured/damaged tissues utilized versatile biomaterials encouraging cell adhesion and proliferation in tissue engineering (TE) [171]. Tissue engineering is a technique for developing biomaterials that can be used to repair, maintain, or regain tissue functionalities or entire organs. Tissue engineering has been a benefit to science. It has the capability of taking the place of traditional treatments, such as xenotransplantation and implanted devices. It is a technique for recovering or regenerating damaged tissues utilizing a combination of scaffolds, cells, and growth regulators. The scaffold components for tissue repair have been developed from various natural polymeric materials. Because of the desirable features, such as good biodegradability, biological activity, and biocompatibility, chitosan has been the most widely recognized biopolymer for constructing tissue frameworks among numerous biopolymers [172]. Table 2 describes the recent studies on chitosan formulations utilized for tissue engineering.

### 11.1. Bone

Bone tissue engineering (BTE) is a rapidly growing subject of research because it circumvents the limitations in therapeutic bone therapies, such as allogeneic or autologous bone transplantation, and irreversible prostheses’ implants [173]. Using scaffolds, biomolecules, and implantable devices, BTE plays a critical role in the healing and regeneration of bone. When the injury is severe and has developed, bone tissue loses its potential to self-heal [174,175,176]. Nevertheless, this technique causes issues such as cell-specific bioactivity, the formation of massive structures, and a less inter-linked system [177]. As a result, nanoparticles and nanostructured materials are used to resolve this issue [178]. Chitosan has been explored for bone tissue regeneration because of its easy fabrication, chemical adjustments, compatibility with tissues, as well as other biomaterials, and its non-toxic nature.

**Figure 4:**
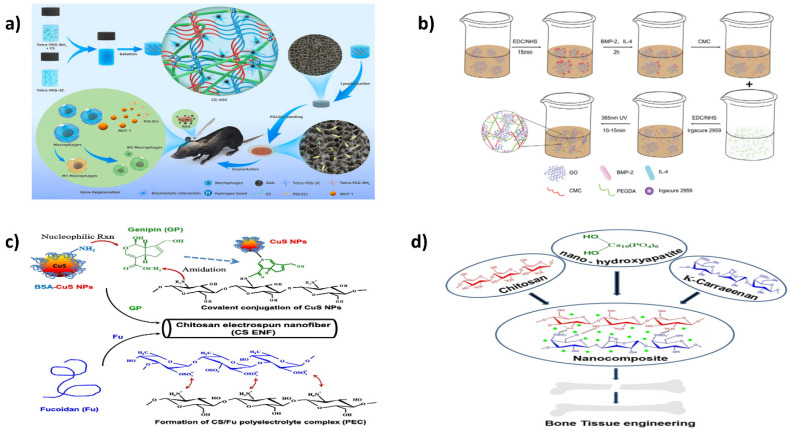
(**a**) Production of a biocompatible composite gel with long-lasting aspirin release for bone tissue regeneration [179]; (**b**) An interpenetrating network hydrogel with a GO-based controlled release mechanism induced M2-type macrophage differentiation for defective bone healing. Reproduced with permission from [183], copyright Wiley Online Library 2021; (**c**) CuS and fucoidan-modified chitosan nanofibers for antimicrobial and bone tissue regeneration. Reproduced with permission from [184], copyright Elsevier 2022; (**d**) Nanocomposite biomaterials for bone regeneration produced from nano-hydroxyapatite impregnated in Chitosan/κ-Carrageenan. Reproduced with permission from [185], copyright Wiley Online Library 2022.

Chitosan is reported to promote cell growth, suppress inflammatory reactions, and cause wound healing. In the early 2000s, Park et al. fabricated CS scaffolds loaded with platelet-derived growth factor (PDGF) for tissue regeneration in rat calvarial bones. Bernardi et al. showed that CS-derived scaffolds act as an independent stimulant for osteogenic maturation and do not merely act as a cell carrier. Nonetheless, native CS hydrogel has insufficient mechanical strength and a tendency for in vivo degradation. Thus, Zhang et al. incorporated short-chain chitosan (CS) into a semi-interpenetrating composite hydrogel (CG) in a covalently bound tetra-armed poly (ethylene glycol) network, as shown in Figure 4a. Acetylsalicylic acid (ASA) was encapsulated in the network by chain intermeshing and electrostatic attraction and obtained sustained release for more than 14 days and promoted osteogenic differentiation and growth of periodontal ligament stem cells (PDLSC) in a mouse calvarial osteogenic-defect model. This could be due to the expression of monocyte chemoattractant protein-1 on host mesenchymal stem cells and PDLSCs, which led to the stimulation of M2 macrophages and in situ polarization, demonstrating its potential for BTE [179].

Biopolymer-based nanomaterials have lately been used in therapeutic applications such as suture components, therapeutic delivery systems, tissue scaffolds, and inner bone stabilization implant devices. The biofilms formed by polymer-derived implants, on the other hand, are very sensitive to microbiological adherence. Chitosan biopolymer has good flexibility but poor mechanical properties; hence, when combined in composite films or nanoparticles, it leads to increased surface porosity and mechanical properties [180,181]. To this end, Prakash et al. created Chitosan/Polyvinyl alcohol/Graphene oxide/Hydroxyapatite/gold film materials for possible orthopedic applications. The graphene oxide/hydroxyapatite/gold hybrid (GO/HAP/Au) was made using a facile hydrothermal process, and the GO/HAP/Au composite integrated polymer film was made using the gel casting process. The biofilms were revealed to be compatible with murine mesenchymal cells (3.74% RBC lysis) and promoted osteoblast development, as indicated by higher alkaline phosphatase enzymatic activity within the cells. As an outcome of these findings, it appeared that the biocomposite films created have osteogenic capabilities for managing bone-related disorders. High mechanical strength was seen, with tensile strength values ranging from 35.2% to 36.4% for composite films. According to the microbiological investigation, these films had high inhibitory regions against gram-positive and gram-negative microorganisms (*Pseudomonas aeruginosa, Staphylococcus aureus, Streptococcus mutans*, and *Escherichia coli*). As a result, the biocomposite biofilms developed were extremely biocompatible and could be employed for bone tissue regeneration [182].

Immune-modifying biomaterials have rapidly evolved as critical new systems for bone tissue regeneration. Eliciting macrophages to develop into the M2 subtype can lower inflammatory and immunological responses and speed the tissue healing following implants. Two biological compounds, bone morphogenetic protein-2 (BMP-2) and interleukin-4 (IL-4), were loaded and delivered in a controlled way in an inter-penetrable network hydrogel made of graphene oxide (GO)-carboxymethyl chitosan (CMC)/poly (ethylene glycol) diacrylate (PEGDA) to stimulate macrophage differentiation into M2 variety and improve bone growth in a study by Zou et al. (as depicted in Figure 4b). These two components were loaded with GO before being incorporated into a CMC/PEGDA hydrogel for long-term delivery. The hydrogel had improved mechanical rigidity, hardness, and stability. In vitro, the hydrogel containing IL-4 and BMP-2 strongly stimulated macrophage M2-type development and bone-marrow mesenchymal stem-cell bone formation. Moreover, in vivo investigations revealed that 8 weeks after insertion, the implantation of this hydrogel significantly lowered the local inflammatory reaction while increasing bone growth. Overall, the findings implied that IL-4- and BMP-2-loaded hydrogels synergistically impact on bone repair. A system such as this for the initiation and immunomodulatory reaction could be a potential method for further bone immunological management and tissue regeneration [183].

Similarly, Lu et al. used electrospun nanofibers of chitosan (CS ENFs) and modified them with fucoidan (Fu) and a CuS NPs polyelectrolyte complex involving genipin-based cross-linking, as shown in Figure 4c. This CuS–ENF composite provided antibacterial effects via photocatalytic and photothermal activities. Moreover, the composite could effectively promote the osteoblastic cells’ alkaline phosphatase activity and the growth of capillary tubes within the endothelium. Thus, this novel CS ENF modification strategy could obtain scaffolds for BTE and antibacterial activity [184].

The foundation for generating nano-hydroxyapatite particles integrated in Chitosan/-carrageenan polyelectrolyte complexes (nHAp/CHI/-CGN) nanostructures with physically cross-linked constituents was presented by Zia and coworkers, as illustrated in Figure 4d. In the SBF investigation, the synthesized hybrid composites were shown to have lattice parameters and tensile characteristics similar to native bone and a rough texture, creating a thick apatite-like covering. It was also cytocompatible, biodegradable, and effective for protein adhesion. The nHAp/CHI/-CGN composites were highlighted as a promising contender for a BTE framework [185].

Similarly, other composite scaffolds that researchers developed include chitosan/decellularized Alstroemeria flower stem [186]; chitosan/gelatin electrospun fibers [187]; chitosan thermo- and pH-responsive hydrogels [182,188]; chitosan/regenerated cellulose nanofibers [189]; copper(II)-chitosan/strontium-substituted hydroxyapatite [190]; chitosan/montmorillonite [191]; chitosan/silver polymeric scaffold [192]; carboxymethyl chitosan/polycaprolactone nanofibers [193]; and chitosan/collagen/hyaluronic acid oligosaccharides [194].

### 11.2. Cartilage

Chondrocytes create the extracellular matrix (ECM) protein molecules found in cartilages [195]. Articular cartilage provides crucial biomechanical activities to bone structures, including abrasion tolerance, load-carrying, and shock attenuation [196]. Since cartilage tissues are usually devoid of blood vessels, have a complicated structure, have quite a small density of cells, and have significant variability, it is harder to treat them [197]. Furthermore, they work in a rigorous atmosphere. So, when the thickness of the damage exceeds 4 mm, the ability for impulsive self-repair is reduced. Mosaicplasty, autologous chondrocyte injections, and micro-fracture are common therapies for cartilage tissue injuries, although they are not always the same structurally as natural tissue [198]. As a result, bioengineering has emerged as a viable option for osteochondral regeneration.

Given its ability to be employed in various ways, such as fibers, sponges, and hydrogels, chitosan is often utilized in cartilage tissue regeneration [15,199,200]. Chitosan’s resemblance to the GAGs present in ECM is also another significant aspect that renders it a desired substance in this domain [201]. Variable electrostatic exchanges with cytokines, receptors, and cell adhesion factors are notable features of GAGs. GAGs can also promote cartilage chondrogenesis. Chitosan can promote chondrogenesis, cartilage-specific protein production, and binding by electrostatic interactions with oppositely charged GAGs [202]. As a result of chitosan’s ability to assist in or encourage the production of cartilage’s unique GAGs, chitosan-derived composite scaffolds have now become attractive for osteochondral regeneration [203,204].

**Figure 5:**
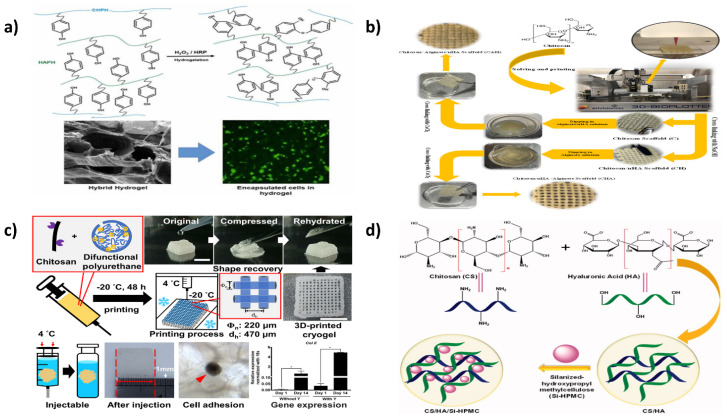
(**a**) Enzymatic synthesizing chitosan/hyaluronic acid hydrogel scaffolds towards cartilage regeneration. Reproduced with permission from [205], copyright Elsevier 2022; (**b**) 3D printing and loading techniques were used to create chitosan/alginate/hydroxyapatite composite scaffolds for prospective cartilage tissue engineering. Reproduced with permission from [206], copyright Elsevier 2022; (**c**) Chitosan cryogel 3D printing as injectable and shape-restorable tissue engineering scaffolds. Reproduced with permission from [207], copyright Elsevier 2022; (**d**) The production steps for the Si-HPMC integrated CS/HA injectable hydrogel are depicted schematically [209].

In the production of multipurpose microhabitats for cultured cells and tissue construction, bioinspired hydrogels are produced. Several mixtures of chitosan (CH) and hyaluronic acid (HA) derivatives with opposing ions were produced in the investigation by Davachi et al. To improve the communication among these constituents, phenolic moieties were supplemented on the backbones of CH (CHPH) and HA (HAPH) through a carbodiimide-based condensation, and further enzyme-assisted cross-linking in the availability of horseradish peroxidase was used to form a robust microenvironment for cell intercalation and tissue regeneration, as depicted in Figure 5a. The hydrogels’ viscoelastic and structural properties revealed that a modest amount of HAPH produces the most remarkable outcomes in the structure. Cellular experiments revealed optimal cell survival and multiplication on the optimal hybrid hydrogel surfaces compared to plain hydrogels. In addition, the composite hydrogels showed better features for developing chondrocyte biomarkers and a greater tendency for MSCs to develop into cartilage-like cells. Altogether, the findings imply that in three-dimensional cartilage tissue regeneration, the optimal composite hydrogel can create an improved biological milieu for chondrocytes [205].

Three-dimensional (3D) printed hydrogel composites containing ceramics have shown promise for cartilage tissue engineering, but their mechanical and biological qualities remain unsatisfactory. In a study by Sadeghi et al. the production of chitosan/alginate-based scaffolds with nano-hydroxyapatite (nHA) using a combination of 3D printing and impregnating processes resulted in a combination, yet new, scaffold architecture for cartilage tissue engineering, as illustrated in Figure 5b. The introduction of nHA raised the Young’s modulus of the scaffolds. In addition, the live/dead assay revealed that nHA significantly impacted the ATCD5 cell adhesion and scaffold survival. Moreover, the scaffolds containing nHA embedded in alginate hydrogels improved the cell survival and adhesion. Additionally, the chitosan scaffolds had good antibacterial properties, which were further increased by the nHA-based scaffolds. Overall, the chitosan/HA/alginate composite scaffolds are potential for cartilage tissue engineering, as well as the methodologies developed to generate hybrid scaffolds using 3D printing and impregnation methods, which could be applied to fabricate scaffolds for other applications [206].

For biological purposes, cryogel offers a highly porous architecture with mechanical strength and injectability. Three-dimensional (3D) printing is a form of customized manufacturing. Unfortunately, there is have been limited investigations into cryogel 3D printing. Chen et al. created a 3D-printable chitosan-based cryogel by employing polyfunctional polyurethane nanoparticles as the crosslinking agent, which interacted with chitosan at 4 °C for 4 h to build a rigid pre-cryogel for 3D printing, as shown in Figure 5c. To make 3D-printed chitosan cryogel, the generated pre-cryogel was frozen at 20 °C. The 3D-printed cryogel had features comparable to bulk cryogel, including high compression, elasticity modulus, and 3200 percent water absorption. The cell tests revealed that the 3D-printed chitosan cryogel frameworks offered good mechanical properties for human adipose-tissue adult stem-cell propagation and chondrocyte differentiation. The injectable and shape-recoverable 3D-printed chitosan-based cryogel scaffolds are viable substrates for tailored tissue regeneration and less surgical intervention [207].

Engineering a multiple-layered chitosan-based scaffold for osteochondral injury healing required a novel method. A highly porous, bioresorbable framework with a unique pore size distribution (mean = 160–275 m) was created by Pitrolino et al. using a hybrid of freeze-drying and porogen-leaching techniques. The inclusion of 70-weight percent nano-hydroxyapatite (nHA) in the bone-like layer strengthened it. The scaffolding displayed fast mechanical restoration during compression loads and did not undergo delamination under tensile load capacity. The scaffold allowed human MSCs to adhere and proliferate, exhibiting adherent cell morphology on the bony layer vs. the spherical cell shape on the chondrocyte layer. In vitro, the unique pore gradients and material constitution favored the osteogenic and chondrogenic development of MSCs in specific layers of the platform. This scaffold offered the ability to rebuild injured bone and cartilage and was an excellent option for noninvasive arthroscopic administration in the clinical setting [208].

When integrated alone without the inclusion of a chemical crosslinker, a new category of composite hydrogels obtained from chitosan (CS)/hyaluronic acid (HA) and silanized-hydroxypropyl methylcellulose (Si-HPMC) (CS/HA/Si-HPMC) have been synthesized and evaluated as injectable hydrogels for cartilage regeneration by Hu et al. (as shown in Figure 5d). The mechanical investigations revealed that, as the amount of Si-HPMC in the hydrogel grew, the swelling ratio and rheological characteristics rose, the compression strength fell, and the decomposition rate accelerated, so that particularly those containing 3.0% (*w*/*v*) Si-HPMC and 2.5/4.0% (*w*/*v*) CS/HA were feasible for cartilage tissue regeneration. The rate of regeneration was around 79.5% on the 21st day of the in vitro studies on cartilage ECM and was suitable for repairing joint cartilage tissue [209].

Other studies for chondral tissue regeneration involve chitosan/xanthan/amniotic fluid stem cells [210], and thiolated chitosan/silk fibroin [211].

### 11.3. Blood Vessel

Artificial grafts, which allow cells to create viable regenerative tissue, are extensively utilized in vascular tissue engineering [212]. Nevertheless, its usability is frequently restricted due to various host-cell invasions, calcification, or inadequate remodeling. Size incompatibility, supply shortage, and previous arterial disorders are all problems with vascular autografts. Because the biomimetic implants or patches are proximate with the bloodstream, they must be non-toxic. Inflammatory responses and calcification, as well as post-surgical loss, might result from incompatibility. Biomaterial deterioration must be regulated so that it does not disintegrate too quickly or inadequately. The transplant is prone to form failure if it deteriorates too quickly. Cell growth and incorporation are hampered if it diminishes too gradually. As a result, effectiveness in blood vascular tissue engineering hinges on developing biodegradable polymer grafts, which can sustain cell uptake and multiplication while also undergoing fast remodeling. Based on its porosity and gel-forming capabilities, chitosan would not just be highly biocompatible and degradable but could simply be conveniently tweaked to show desirable qualities. Moreover, chitosan is a glucosamine and an N-acetyl glucosamine biopolymer. Glycosaminoglycans, having a striking resemblance to chitosan, make up the ECM of vasculature tissue. Mixtures of chitosan and other polymeric materials have demonstrated promising improvements in structural rigidity, cell attachment, and growth in this setting [212,213,214].

Wang et al. made a hybrid scaffold out of gelatin methacryloyl (GelMA) and carboxymethyl chitosan (CMCS), demonstrating its ability to stimulate revascularization. Compared to the pure GelMA scaffold, the composite GelMA/CMCS scaffolds implanted with BMSCs displayed outstanding mechanical characteristics, CD31 induction, and expression of vasculogenic genetic factors. The BMSC function in 3D-printed GelMA/CMCS scaffolds also revealed the potential of bioprinting cells impregnated with GelMA/CMCS hybrid scaffolds. Thus, GelMA/CMCS hybrid scaffolds have the potential to be used in vascular tissue regeneration [215].

Because a discrepancy in mechanical parameters between vascular patches and natural blood vasculature might lead to post-operative failures, vascular patches that mirror the biomechanics of natural blood capillaries should be designed. They created a bioinspired vascular patch by treating a decellularized scaffold (DCS) with a poly (ethylene glycol) (PEG) membrane and altering its surfaces with a heparin–chitosan polyelectrolyte multilayer (PEM). The PEM-functionalized PEG/DCS vascular patches had mechanical properties similar to natural blood vessels. They efficiently repelled platelet attachment, lowered hemolysis, enhanced coagulation time in vitro, and encouraged endothelial cell attachment and development. Additionally, the customized patches preserved the arteries’ patency for an extended period (5 months) in vivo [216].

An electrospinning procedure was employed to build a scaffold for blood vessel regeneration using Poly-L-Lactic acid (PLLA) combined with chitosan and collagen in a study by Fiqrianti et al. The conduit’s shape, chemical bonding, compressive strength, bursting pressure, hemocompatibility, and cellular viability were all studied using different amounts of chitosan in the mixture. In vitro tests revealed that adding chitosan–collagen to a cell culture could enhance cell survival and hemostasis. The conduit containing 10% PLLA, 0.5 percent chitosan, and 1 percent collagen had optimal results. The compressive modulus was 2.13 MPa, and the burst pressure was 2593 mmHg, both of which were within the biological blood vessel parameters threshold. The vascular graft component met the requirements of high hemocompatibility and lower toxicity with a hemolytic rate of 1.04 percent and a survival rate of 86.2 percent. The findings are encouraging for future research into vascular graft applications [217].

In a study by Soriente et al., they investigated how well the multifunctional chitosan (CS)/Poly (ethylene glycol) diacrylate (PEGDA) related scaffolds might promote revascularization in vitro. The substrates were bioactivated using both organic (BMP-2 peptide) and inorganic (hydroxyapatite nanoparticles) stimuli. In vitro angiogenic assays, focused on cell growth and differentiation, were used to investigate the qualities of the substances regarding physiological response stimulation on human umbilical vein endothelial cells (HUVECs). Their findings showed that the functional signals on the top of the CS/PEGDA scaffolds positively impacted angiogenesis responses, as measured by angiogenic marker production (CD-31) and endothelial tissue development (tube generation). Thus, the bioactive CS/PEGDA scaffolds could be new inserts for encouraging the angiogenesis of tissue-engineered constructions in the domain of tissue engineering [218].

The grafting failure of medically useful synthetic tissues is caused by defects in the creation of microvascular systems, which bring oxygen and other nutrients to the cells. The vascularization of synthetic tissues can be aided by inflammation and immunomodulatory reactions. Endothelial progenitor cells (EPCs) and RAW264.7 macrophages were used as model cells in a capillary structure made of a gelatin methacrylate-derived cell-laden hydrogel scaffold complexed with interleukin-4 (IL-4)-loaded alginate-chitosan (AC) microspheres. Through electrostatic attachment, the AC microspheres retained and directed the EPCs, allowing for the creation of microvascular connections. The IL-4-loaded microspheres encouraged macrophage polarization into the M2 type, which resulted in a decrease in pro-inflammatory markers and an increase in vasculature [219].

### 11.4. Corneal

Ocular tissue engineering is a vital branch of bioengineering. The corneal epithelium oversees keeping the cornea clear by pushing ophthalmic fluid into the eyes. Since human corneal epithelial cells cannot renew, visual impairment occurs when they are lost due to aging, injury, or illness. Blindness develops when the body’s cells decrease dramatically [220]. According to the World Health Organization, corneal degeneration and cataracts are among the reasons for visual impairment worldwide [221]. Corneal tissue engineering for retinal cell therapy is crucial because of the growing market for, and scarcity of, corneal donations.

The corneal tissue is a non-vascularized tissue that provides visibility. Corneal wounds are frequently repaired with amniotic membrane transplantation, which can be infected and immuno-rejected [222]. In the realm of cornea tissue regeneration, scaffold or membranes created from chitosan, gelatin, genipin, and other polymers are being investigated. Chitosan is popular because of its cytocompatibility and anti-inflammatory properties; however, its scaffolds have limitations, such as low structural rigidity. Polymers are combined with chitosan to improve scaffold qualities, similar to those of existing tissue engineering applications. Cornea tissue supports should have tensile and visual features identical to those of the corneal membrane and the capacity to sustain cells and adhesiveness. Because optical clarity is a critical quality for corneal prostheses, special care must be taken while choosing the material and production procedures. Under surgical treatment, the scaffolds or films should also be malleable and adaptive. The amniotic membranes now employed in clinical practice are not thick enough, disintegrate quickly, and have hygienic storage concerns [223,224].

By integrating chitosan nanoparticles (CSNPs) into chitosan/polycaprolactone (PCL) films, Tayebi et al. created a biodegradable transparent framework for growing corneal endothelium using the solvent casting process. The CSNP/PCL ratio increased, enhancing clarity and surface water sorption. The CSNP/PCL 50/25, which has the lowest WCA, demonstrated similar transparency with human acellular corneal stroma. According to the MTT experiment, the scaffold was non-cytotoxic and enhanced HCEC development. The HCECs were adequately attached to the framework and produced a dense monolayer. From the perspective of transparency and cytocompatibility, the scaffold is appropriate for corneal endothelium repair [225].

Clear, biologically compatible, and in situ formed biomimetic substrates are ideal for ocular bioengineering because they can profoundly fill irregularly shaped corneal stroma abnormalities and facilitate tissue repair. To this end, Feng et al. created a new class of corneal frameworks using oligoethylene glycol (OEG)-based dendronized chitosan (DCs), which have fascinating sol–gel shifts induced by temperatures close to physiological conditions, culminating in very clear, translucent hydrogels. These hydrogels’ gelation points can be easily adjusted, and their tensile performance can be significantly increased when introduced into PBS at 37 °C rather than in pure water. In vitro experiments showed that these DC hydrogels have excellent biocompatibility and could enhance keratocyte differentiation and proliferation. In situ-produced DC hydrogels benefitted potential tissue repair in rabbits’ eyes with cornea stromal deficiencies. Given their high biocompatibility and exceptional thermo-responsiveness, these thermo-gelling DCs have a lot of potential as ocular tissue replacements [226].

A hybrid membrane was created using carboxymethyl chitosan (CMCTS), gelatin, and hyaluronic acid in a study by Xu et al. Primary rabbit corneal epithelial cells (CEpCs) were implanted on it, and it was observed to be translucent, biodegradable, and ideal for CEpC adhesion and growth, as well as maintaining CEpC synthesis of epithelial cell-like proteins. The CEpCs/CMCTS membranes were utilized to treat the alkali-induced corneal injury in rabbits, and the membrane had the potential to enhance corneal epithelial restoration dramatically and regain corneal clarity and thickness [227].

There is no appropriate scaffold for transplanting limbal stem cells (LSCs) into the cornea to stimulate corneal regeneration following corneal alkali-induced burns. To this end, Xu et al. created a new alginate-Chitosan hydrogel (ACH) for LSC implantation in situ. Periodate-involved sodium alginate oxidation yielded sodium alginate dialdehyde (SAD), a physiological crosslinker. SAD quickly crosslinked carboxymethyl chitosan via Schiff’s base reaction between the accessible aldehyde and amino residues. Self-crosslinking causes the ACH to develop rapidly on the wound surface without requiring any chemical cross-linking components. The in situ hydrogel was found to be remarkably transparent, gelled rapidly, bio-friendly, and noncytotoxic. The stem marker p63 was displayed by LSCs cultivated in vitro, while the differentiated epithelial biomarkers cytokeratin 3 and 12 were not. Moreover, the hydrogel encasing LSCs was proved to dramatically increase the epithelium restoration when applied to an alkali-induced burn site on the corneal region. Altogether, such an innovative in situ hydrogel-LSC grafting approach could be a quick and efficient way to cure corneal wounds [228].

Shahin et al. created a chitosan/gelatin hyaline film containing NHS and EDC crosslinking agents to transplant the corneal epithelial cells. Before crosslinking, the two gelatin and chitosan solutions were uniformly combined in proportions of 20/80, 30/70, 40/60, and 50/50 (Gel/Chi). They were dried in the oven for 24 h. It was found that rising chitosan concentrations enhanced the transparency and cytocompatibility of generated samples, greater water penetration and degradation rate of samples, and dramatically improved cell growth and vitality [229].

Other similar studies include chitosan/polycaprolactone [230]; carboxymethyl chitosan/gelatin/potassium acetate [231]; and chitosan/keratocyte spheroids [232].

### 11.5. Periodontal

With its biological properties, antibacterial properties, cytocompatibility, and capability to integrate with other substances, chitosan is evaluated as a viable contender for dental purposes. It works just as well as a single element and, in many circumstances, outperforms it when paired with other synthetic or natural components [75]. Chitosan-based materials are employed in a variety of applications, including enamel remineralization and development [233], dentin bonding [234], tooth repair material [235], and surface coatings for dental implants [236].

Meanwhile, periodontal diseases are among the critical challenges in medical research since it causes irreparable erosion of periodontal tissues, resulting in loss of teeth. In the advent of periodontitis, an inflammatory response condition, the hostile oral microenvironment becomes even more unfavorable. As a result, scientists have been looking for a viable biomaterial substitute. Various types and mixtures of chitosan have already been explored and examined; however, very few publications have been published in the last ten years. Many studies focused primarily on chitosan, while others looked at its ability to mix with several other organic and inorganic compounds. In most cases, the resulting material displayed potential dental action [53].

Chitosan, a promising polymer, is now being utilized in dental implant coating. Nevertheless, there is a paucity of studies on coating materials for implants apart from commercially purified titanium. As a result, Alnufaiy et al. studied the impact of chitosan with two degrees of deacetylation (DDA) as coverings for laser surface microtopographic implants. In the chitosan-functionalized samples, the production of osteogenic biomarkers increased significantly. A high DDA of chitosan aided an enhanced bone mineralization and osteoblast development. As a result, the mixture of laser surfaces and chitosan may improve dental implant recovery and osseointegration procedures [237].

Given its superior biocompatibility, biodegradation by naturally occurring enzymes, suitable physicochemical attributes, and optimum molecular size, chitosan can be employed as a framework for the healing of periodontium (in the therapy of periodontal pockets). Furthermore, pluripotent dental mesenchymal stem cells, including stem cells from human exfoliated deciduous teeth (SHED) and human periodontal ligament cells (HPLCs), can be seeded into chitosan frameworks. Chitosan could be utilized to efficiently regenerate periodontal tissue by stimulating cementoblasts and osteoblasts to produce new tissues. Sukpaita et al. in their study using chitosan/dicarboxylic acid (CS/DA) with seeded HPLCs, found that within 6–12 weeks, significant in vivo bone growth was observed in calvarial-defects mice models [238].

The root canal network is chemo-mechanically debrided, and inflammatory or decaying pulp contaminated by bacteria is removed during root canal therapy. Several studies on the antimicrobial property of chitosan nanoparticles (CSNPs) towards pathogens such as P. gingivali, S. mutans, and E. faecalis have been previously reported. CSNPs could be used with calcium hydroxide as endodontic sealants or for temporary root canal filling. Regenerative endodontic therapies have also used chitosan. Bioactive compounds and growth stimulators can be added to chitosan-derived porous scaffolds. Dentin sialophosphoprotein, alkaline phosphate, and dentin matrix acidic phosphoprotein are odontoblastic indicators that promote the synthesis of secreted signaling chemicals, causing dental pulp stem cells (DPSCs) to proliferate and differentiate into odontoblasts [239].

Similarly, some other studies for dental tissue engineering composed of chitosan-based scaffolds are chitosan/PNIPAAm/graphene oxide [240]; chitosan/Ca-SAPO-34 monometallic or bimetallic nanoparticles [241]; chitosan/calcium [242]; chitosan/nanofluorohydroxyapatite or nanohydroxyapatite [243]; chitosan/gelatin/nanohydroxyapatite [244]; chitosan/PLA-nanofibers [245]; chitosan/collagen/bone morphogenetic protein-7 [246]; chitosan/gelatin [247]; chitosan biguanide/carboxymethyl cellulose [248]; and chitosan/polyurethane nanofibrous membrane/AgNPs [249].

### 11.6. Miscellaneous

#### 11.6.1. Skin

The skin represents the physical barrier between the surrounding environment and the physiological body [250,251]. Damage to the skin involves conditions such as burns, infections, and acute and chronic disorders such as psoriasis [252,253,254,255]. In such instances, TE provides advantages over conventional treatments because of its superior efficiency, fewer chances of donor morbidity, and immuno-compatibility reactions [256]. Additionally, the TE based on biocompatible biomaterials and their composites offer a substitute for fabricating tissue scaffolds that are physiochemically and biologically identical to natural tissues [257,258]. An essential requirement in skin TE is designing novel biopolymeric films or scaffolds resembling the extracellular matrix by combining biological, material chemistry principles and engineering [182], displaying features such as biodegradability, biocompatibility, and material characteristics [259].

Contemporary regenerative therapy is concerned with hypoxia and sepsis. The goal of oxygen-generating biopolymers with antibacterial properties is to address these issues. Oxygen deprivation at the implantation surface causes superoxide radicals, which slow the healing process. In addition, sepsis in the wound leads to a delayed healing process. As a result, antimicrobial and oxygen-producing scaffolds have demonstrated their ability to aid wound healing. The oxygen-releasing, ciprofloxacin-encapsulated collagen-chitosan scaffold used in this work was constructed for long-term oxygen supply. Biochemical oxygen was provided by calcium peroxide (CPO). The oxygenation pattern showed a consistent diffusion of oxygen together with homogeneous CPO accumulation on the scaffold. Ciprofloxacin was released in a sustained manner. Cell culture experiments showed that the scaffold has good cell adhesion and motility capabilities for the fibroblasts. In the in vivo investigations in the skin, the flip model revealed that wound healing was improved, and necrosis was reduced. Histopathological investigations revealed that tissue structure was preserved, and collagen was deposited. The findings indicated that the suggested CPO-coated ciprofloxacin-based collagen–chitosan scaffold could be a suitable skin tissue regeneration alternative [260].

The tissue regeneration capability of the nanofibrous framework incorporating proteins and polysaccharides appears promising. Hence, Mohamad Pezeshki-Modaress investigated the influence of chitosan in chitosan/gelatin nanofibrous scaffolds created using an improved electrospinning method. The culture of dermal fibroblasts (HDF) on nanofibers in respect of adhesion, shape, and growth was investigated to see how the chitosan concentration affected the bioactivity of the electrospun chitosan/gelatin scaffolds for tissue regeneration. According to morphological observations, HDF cells were adhered to and propagated successfully on extremely porous chitosan/gelatin nanofibrous scaffolds with spindle-like forms and stretching. Electrospun gelatin/chitosan scaffolds in culture media kept their fibrous morphologies for 7 days. The MTS assay was used to measure cell proliferation on electrospun gelatin/chitosan scaffolds, revealing a beneficial influence of chitosan concentration (about 30%) and the nanofibrous pattern on scaffolds’ biocompatibility (differentiation and adhesion) [261].

#### 11.6.2. Cardiac Tissue

Injectable biomaterials are a viable therapeutic method for cardiac tissue repair in the treatment of myocardial infarction-related chronic heart failure. Because of the ionized amino acid moieties, chitosan exhibits mucoadhesion, is hemostatic, and is effective in attaching to cellular membranes. Chitosan can also be used to create well-connected scaffolds with enough porosity to ensure cell survival by providing a constant oxygenated blood and nutrient supply [262]. Controlled release of loaded bioactive substances and growth factors becomes another essential aspect of a chitosan-derived scaffold. This makes it an excellent choice for cardiac tissue regeneration. Chitosan is a biocompatible platform that works as an ECM, allowing immobilized angiogenic growth factors to drive endothelial cell migration and proliferation, allowing for the development of a new vasculature to be facilitated [263]. The pig ECM is cross-linked with genipin and chitosan, according to investigations. This aids in maintaining ECM physiological components while also increasing the injected scaffolds’ tensile stability. Before employing non-clinical ECM as a scaffold material for tissue engineering, it must be decellularized to minimize the hypersensitivity of the material [262].

#### 11.6.3. Connective Tissue

The 3D printing of the chitosan hydrogel is difficult because of its poor mechanical properties and weak formation capability. To this end, Zhang et al. fabricated acrylate substituted (DS 1.67) maleic chitosan (MCS) and thiol-terminated poly (ethylene glycol) (TPEG) through a step-chain growth photolytic polymerization technique, which helps in overcoming the formidable oxygen inhibitory effect. A strong intermolecular interaction between MCS and TPEG, and as compared to unmodified chitosan hydrogel, the prepared hydrogel showed a 10-fold and 2-fold enhancement in compression strength and gelling rate, respectively. Thus, 3D-printed chitosan hydrogel could be prepared by concurrent extrusion deposition and acrylate-thiol photopolymerization, having good printing efficiency and enhanced stability of the scaffold. The 3D-printed hydrogel demonstrated no cytotoxicity and supported L929 cell growth [264].

So far, only a handful of studies have been undertaken using carboxylated chitosan as a biomaterial for producing porous scaffolds and gels; hence, Yang et al. developed soft chitosan hydrogels in a hybrid composite with recombinant human collagen (RHC-CHI) through crosslinking-induced gelation method for use as soft-tissue scaffolds for tissue regeneration. They showed tunable mechanical properties by adjusting either the polymer amount or the RHC-to-chitosan ratio. Beyond a specific concentration, increasing chitosan’s concentration decreased the hydrogel’s tensile strength and started causing degradation. The prepared hydrogels were non-cytotoxic and promoted the adhesion and growth of NIH-3T3 cells. The in vivo tests also revealed that the hydrogels could rapidly infiltrate cells and cause wound closure; thus, they are good candidates for soft-tissue regeneration [265].

**Table 2:** Recent studies focusing on tissue engineering applications of chitosan formulations.

Device Type	Model Drug/Drug	Polymer Formulation	Preparation Method	Tissue	Effects/Results	Reference
3D-Nanofibrous scaffold	-	Poly(vinyl alcohol)/keratin/chitosan	Layer-by-layer electrospinning	-	5% *w*/*v* keratin and 2% *w*/*v* chitosan electrospun with 10% *w*/*v* PVA showed remarkable properties such as high tensile strength, which doubled with increasing polymer concentration from 10 wt.% to 50 wt.%, swelling ratio (over 100%), porosity (82% to 86%). However, after 4 weeks of incubation, the scaffolds degraded significantly (50–66%).	[266]
Modified halloysite nanotube-based nanocomposite films	-	Chitosan/PVA/PVP	Solution casting	-	The synthesized nanocomposite films demonstrated enhanced thermal and mechanical attributes, uniform size distribution, surface topology, and enzymatic breakdown, with low swelling ratio and hydrophilic properties. In vitro, MTT and AO-EB assay revealed superior cell proliferation and adhesiveness as compared to neat PVA/PVP films ((118.31 ± 0.68% proliferation by 5 wt.%), and their hemocompatibility with RBCs was low (0.46 ± 0.05%).	[267]
Membranes	-	Chitosan/collagen/hydroxyapatite	Solvent casting	Bone/cartilage	Micro- and nanoporous membranes had excellent hydroxyapatite dispersion in the matrix. Thermally stable composites due to the incorporation of hydroxyapatite and collagen. No cytotoxicity and the highest adhesion were found in the membrane with 1.5% *w*/*v* Cs. 0.75% *w*/*v* collagen and 0.75% *w*/*v* hydroxyapatite.	[268]
Multilayer scaffold		Chitosan/gelatin/nano-hydroxyapatite	Iterative hierarchical method	Bone/cartilage	Adipose mesenchymal stem cells (ADSCs) differentiated into osteoblasts and chondrocytes similar in morphology to natural tissues, facilitating the expression of both osteogenic genes (OCN, Col I, and Runx2) and chondrogenic genes (ACAN, Sox9, and Col II).	[269]
Electrospun nanofibers	-	Chitosan/polypyrrole/collagen	Electrospinning	Heart/nerve/cardiovascular/skin	10% *w*/*w* polypyrrole-containing scaffolds exhibited optimum mechanical properties, good cell attachment, growth, and differentiation.	[270]
Composite scaffold		Chitosan/polyvinyl alcohol/cellulose nanocrystals (CNC)/β-Tricalcium Phosphate	Freeze drying	Bone	5% and 10% of CNC-based scaffolds exhibited significant calcium deposition after 72 h of culture.	[271]
Hydrogels		Chitosan oxidized quince seed gum/curcumin loaded-halloysite nanotubes	Sonication	-	CS/O-QSG (25:75) exhibited rapid gelation and compression strength, and with 10–30%, CUR-HNTs enhanced cellular growth and proliferation by 150%.	[272]
Hydrogels		Chitosan/mucin/Montmorillonite/hydroxyethyl methacrylate	Freeze drying	-	Good material characteristics such as porosity and water uptake as well as biocompatible with C2C12 and MC3T3E1 cell lines.	[273]
Films		Chitosan/collagen	Dual crosslinking with genipin and tannic acid	Cornea/skin	80% film retention after 2 weeks of incubating with lipase and lysozyme and biocompatible with mouse fibroblast cells.	[274]
Films		Chitosan/silk fibroin	Solvent casting	Bone/adipose/cartilage/skin	Excellent adhesion, growth, and proliferation of rat bone-marrow-derived mesenchymal stem cells, while promoting osteogenic and adipose differentiation.	[275]
Hydrogels		Chitosan/decellularized annulus fibrosis matrix (DAFM)	Freeze drying	Intervertebral disc	Sustained release of fibroblast growth factors as a stimulus for AFSC growth and expression of ECM factors.	[276]

## 12. Wound Healing

Apart from its applications in drug delivery and tissue regeneration, some antifungal and antibacterial activity have also been discovered with chitosan and its quaternary derivatives [277,278]. It offers additional benefits compared to other synthetic derivatives, such as its higher killing efficiency, broader antibacterial spectrum, and lower mammalian toxicity [279].

Modifications of chitosan with amino acids have had tremendous advantages for wound healing applications. Several approaches may be carried out for modifying native chitosan with amino acids, including physical and chemical methods, such as like blending, compositing, grafting, cross-linking, etc. Since the amino groups of chitosan have two distinct types of properties, alkaline as well as nucleophilic characteristics, chitosan can form imines and amides and can also lead to the formation of salt derivatives. It has been reported that the functionalization of chitosan with amino acids offers enhanced cell adhesiveness, re-epithelialization potential, rapid angiogenesis, and collagen formation [280].

**Figure 6:**
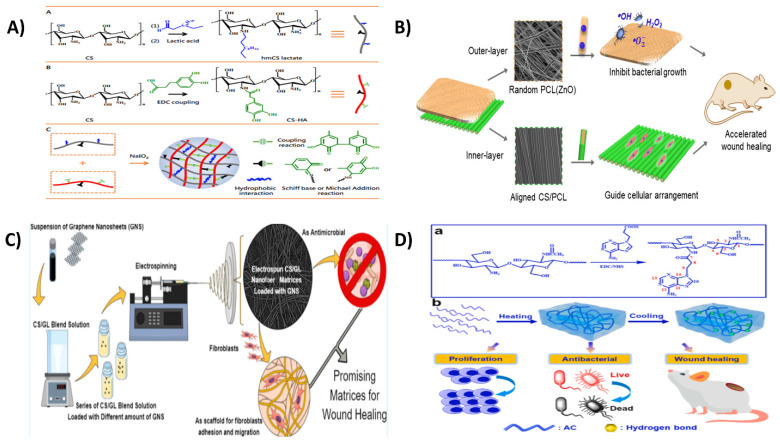
(**A**) Sutureless wound closure with anti-infective and pro-coagulator chitosan-mediated hydrogel tissue adhesives, reproduced with permission from [281], copyright ACS publications 2020; (**B**) Enhanced wound healing with electrospun ZnO-impregnated chitosan/PCL bilayer membranes featuring a spatially tailored structure. Reproduced with permission from [284], copyright Elsevier 2022; (**C**) Graphene-reinforced electrospinning-based chitosan/gelatin nanocomposite scaffolds having antibacterial and wound-healing properties [285]; (**D**) Chitosan-based hydrogels for wound repair that are injectable, self-healing, and antimicrobial. Reproduced with permission from [286], copyright Elsevier 2022.

Chitosan alone has quite limited tissue adhesiveness in a wet environment; hence, catechol-based chitosan was explored because of its ability to form strong covalent interactions between the tissue’s thiol or amino group and oxidized catechol moieties. The catechol-functionalized chitosan also showed good anti-infective and tissue adhesive activity. To preserve the anticoagulant effect of chitosan, hydrophobically modified chitosan (hmCS) consisting of alkyl side chains was used as an amphiphilic analog of chitosan. Du et al. developed a hmCS lactate, and hydrocaffeic acid-modified chitosan (CS-HA)-based hydrogel via a two-step procedure, as shown in Figure 6A, which demonstrated good anti-infective activity against *P. aeruginosa* and *S. aureus*. The CS-HA/hmCS hydrogel was non-cytotoxic to 3T3 fibroblast cells and was capable of sutureless wound healing in a rat full-thickness skin model due to the remarkable tissue interfacial adhesiveness of the optimum Gel3, which could maintain the incision structure and promote wound healing [281].

In civil and wartime situations, producing an anti-infective shape-memory hemostasis sponge capable of guiding in situ tissue repair for noncompressible hemorrhages represents a problem. Chitosan has applications in producing hemostats because of its anti-infectivity, biocompatibility, non-toxicity, hemostasis, and so on [282]. However, in situations where severe hemorrhage or microbial infections occur, its hemostatic potential is quite limited [281]. To this end, Du et al. used a combination of 3D-printed microfiber leaching, freeze-drying, and surface-active modifications to create hemostatic chitosan sponges featuring strongly interconnective micro-channels. They showed that the micro-channeled alkylated chitosan sponge (MACS) could absorb water and blood while also restoring its structure quickly. In potentially deadly, healthy, and heparinized rats and pig hepatic-perforated wound specimens, the MACS provided better pro-coagulant and hemostatic capabilities than the clinically utilized gauze, gelatin sponge, CELOX^TM^ (Crewe, UK), and CELOX^TM^ gauze. In a rat liver damage model, they showed that it has anti-infective efficacy against *S. aureus* and *E. coli* and promotes liver parenchymal cell migration, vasculature, and tissue incorporation. Therefore, the MACS showed promise as a therapeutic translational tool for treating deadly noncompressible bleeding and promoting healing [283].

Similarly, Zhou et al. created a versatile multilayer membrane with electrospun chitosan (CS) and activated ZnO nanoparticles (as illustrated in Figure 6B). The bilayer membrane’s external surface was made up of ZnO-loaded poly(-caprolactone) (PCL) fine fibers in an irregularly oriented arrangement, giving it many antimicrobial properties. The internal layer consisted of CS fibers with a core design that might perform as an anti-inflammatory and efficient cell interaction guide. Notably, the composite CS/PCL electrospun membranes containing 1.2 wt. percent ZnO nanoparticles had improved mechanical properties and a clear inhibiting zone against *E. coli* and *S. aureus*, as well as being non-cytotoxic to fibroblast cells. In addition, the bi-layered membranes allowed for the attainment of substantial ZnO nanoparticle bioavailability and coordination with the oriented structural characteristic of CS fibers, which reduced inflammation, encouraged cell motility, and allowed for re-epithelialization in vivo [284].

Ali et al. created electrospun chitosan/gelatin nanofibrous scaffolds that were strengthened with various amounts of graphene nanosheets and could be employed as antimicrobial and wound-healing constructs, as shown in Figure 6C. The different manufactured scaffolds were fully characterized before being tested for antibacterial activity, cytotoxicity, and cellular migration potential against *Escherichia coli* and *Staphylococcus aureus.* Nanostructures combined with 0.15 percent graphene nanosheets had the smallest width (106 ± 30 nm) and the most significant porosity (90 percent), along with good renewability and swellability. Nevertheless, increasing the number of graphene nanosheets by too great a degree resulted in beaded nanofibers with lower porosity, swelling ability, and degradability. Nanostructures reinforced using 0.15 percent graphene nanosheets, on the other hand, inhibited *E. coli* and *S. aureus* development by 50 and 80 percent, accordingly. When adult fibroblasts were cultivated with either non-reinforced or reinforced nanomaterials, the in vitro cytotoxicity experiments revealed negligible damage. After 24 and 48 h, cell movement was greater in the strengthened nanofibers compared to unmodified nanofibers, which is due mainly to the significant impact of graphene nanosheets on cellular migratory capability. After 48 h, cell migration outcomes for reinforced and unreinforced nanofibers were up to 93.69 and 97 percent, correspondingly [285].

A crucial healthcare concern is developing a cost-effective and readily available substance for better skin tissue regeneration. Hence, Deng et al. created injectable, self-healing adenine-functionalized chitosan (AC) hydrogels meant to expedite the healing process significantly without the need for medicinal agents and were inspired by the notion of wet wound repair, as shown in Figure 6D. In aqueous solutions, various AC derivatives with degrees of substitution (DS) ranging from 0.21 to 0.55 were synthesized, and AC hydrogels were constructed using a facile heating/cooling technique. AC hydrogels showed remarkable self-healing capability, low swelling rates, cytocompatibility, cell proliferation promotion, and hemostatic action. The hydrogels were proved to possess antibacterial properties against gram-negative and gram-positive bacteria, fungus, and bacteria with antibiotic resistance. Furthermore, full-thickness skin lesion model investigations revealed that AC hydrogels could greatly minimize inflammatory cell invasion and speed the wound repair. The hydrogel has the potential to revolutionize the design of multipurpose wound dressings [286].

Similar studies exploring the antibacterial wound healing abilities of chitosan were chitosan-based quaternary ammonium salt/gentamicin sulphate hydrogel films [287]; chitosan/polyvinyl alcohol hydrogel/ZnO nanoparticles [288]; chitosan/pectin/lidocaine hydrogel [289]; quaternized chitosan/Matrigel/polyacrylamide hydrogels [290]; chitosan/carboxymethyl chitosan/AgNPs polyelectrolyte complex [291]; chitosan/PVA/starch electrospun mats [292]; carboxymethyl chitosan/polyurethane/helatin hydrolysate [293]; oxidized chitosan/amidated pectin hydrogel [294]; chitosan/PVA/copper [295]; and chitosan/PVA/HKUST-1 electrospun mats [296].

Table 3 depicts the latest investigations on chitosan formulations for wound healing

Despite its various advantages, chitosan and chitosan-based formulations do suffer various drawbacks. The variations due to the source and preparation methods of chitosan have a direct influence on their mechanical and biological properties [307]. Moreover, due to its high solubility at an acidic pH, the ability of chitosan to control the release rate and stability of drugs is limited, requiring an additional coating of acid-resistant anionic polymers, such as alginate [308]. In gene delivery applications, the transfection efficiency of chitosan as a non-viral carrier depends on the media pH, degree of acetylation, cell type, molecular weight, etc. Most reports focus on the in vitro gene delivery efficacy, however, extensive research on in vivo models is still scarce [309].

## 13. Conclusion and Future Perspectives

Chitosan displays remarkable physicochemical attributes and good biocompatibility and links with human proteins, cells, and organs. The terminal amino groups present in its skeleton facilitate the formation of polycations in an acidic medium when the amine groups undergo protonation; this enables interactions with anionic polymers in various shapes and geometry. It is expected that chitosan and chitosan-based derivatives may be used to fabricate tissue repair scaffolds for a plethora of tissues, such as bone, cartilage, skin, cornea, blood vessel, and so on, with suitable properties. Such matrices are beneficial because of their natural resemblance to host tissues and similarities in structure and function to biological molecules.

As humans become more aware of the intricate biological reactions to the presently available biomaterials, as well as with the expanding knowledge about the human anatomy and physiology, organ and tissue damage, disease proliferation, and cellular changes in protein functions during tissue injury, a co-operative endeavor involving polymer chemists, engineering minds, biologists, and physicians could be undertaken for developing novel polymeric biomaterials specially tailored for each application. Currently, chitosan stands as one of the most reliable and convenient biomaterials for various kinds of biomedical applications, mainly due to its accessibility and peculiarities. Nonetheless, added efforts need to be taken to enhance the tissue scaffold for tailored properties for different types of tissues. Along with in vitro studies, the same scaffolds must also be tested in vivo to determine their clinical utility in humans. Several studies have shown that the in vitro benefits of chitosan composites for tissue and wound healing do not translate as expected in the in vivo animal models. A closer look into the reasons and modifications required must be encouraged.

With the remarkable progress of tissue engineering science such as novel stem cell sources, microfluidics’ devices, versatile and tailored biomaterials, etc., some challenges remain, such as engineering blood vessels into the tissue scaffolds, the immunogenicity of the scaffolds, and regulatory issues. The 3D printing platform provides the flexibility to print complex structures, such as cells and scaffolds, while also giving good control of pore size and size distribution. Consequently, recent publications depict the utility of chitosan in printing constructs for bone, skin, vascular, and cartilage tissue engineering. However, their practical applications and clinical translation are still being investigated. Recent reports have suggested that chitosan and its quaternized derivative can have immunomodulatory effects by activating the antigen-presenting cells and inducing cytokine production. Hence, their role as vaccine adjuvants could open new paradigms in vaccine formulation delivery.

Although there have been definitive strides in technological advancements, more profound research is warranted for evaluating cell-specific intercommunications, in vivo understanding, and replication of bioactivity, long-term stability, and biocompatibility studies to make chitosan a more widely used biomaterial.

## Figures and Tables

**Table 1:** Recent studies on chitosan focusing on delivering therapeutic compounds.

Device Type	Model Drug/Drug	Polymer Formulation	Preparation Method	Administration Route	Delivered Site	Effect/Results	References
Solid–lipid nanoparticles	Leflunomide	Chitosan/Folic acid	Layer-by-layer coating	oral	joint	FA-CS-SLNs exhibited sustained release for 168 h and lowered liver toxicity and enhanced joint healing compared to leflunomide suspensions.	[161]
Implants	Ibuprofen	Chitosan/polycaprolactone	Hotmelt extrusion/Fused deposition modeling	-	-	Sustained release for 120 h by diffusion-erosion, 75.3% cell viability.	[162]
Hydrogel-based microneedles	Salvia miltiorrhiza	Carboxymethyl chitosan/oxidized pullulan		Skin	Mucosa	Simple penetration of HFM-1 into infant porcine skin was demonstrated because of its remarkable mechanical characteristics. Drug release was rapid from HFM-1, hence suitable for transdermal delivery.	[163]
Hydrogel	Berberine chloride hydrate	Chitosan/puerarin	Interpenetrating network	Injectable		Increased rheological properties due to dense structure of CS/PUE_18_ hydrogels. Dual anti-inflammatory and antimicrobial activities with pH-dependent drug release	[164]
Thermogel	Dexamethasone	Hexanoyl glycol chitosan		Injectable	Inner ear	Versatile release kinetics with no initial burst release. Excellent residual stability without any ear-related side effects and could deliver high concentrations of the drug to the inner ear.	[165]
Hydrogel	Acyclovir	Chitosan/β-cyclodextrin/methacrylic acid (MAA) and N′ N′-methylenebis-acrylamide (MBA)	Free radical polymerization	Oral		Zero-order kinetics of drug release with pH-dependent swelling behavior. After acute oral toxicity studies, no significant behavioral, histopathological, and clinical changes were observed in Wistar rats. Increased bioavailability as compared to acyclovir suspension at a dose of 20 mg/kg in rabbit plasma.	[166]
Hollow capsule	Gemcitabine/curcumin	Chitosan/poly(ethylene glycol dimethacrylate-co-methacrylic acid)	Layer-by-layer method			pH-dependent drug delivery at pHs 5.5 and 7.4. Encapsulation efficiency was above 84%, and release efficiency was 82%. Good cytotoxicity towards HCT-116 colorectal cancer cell lines.	[167]
Nanohybrid	5-fluorouracil	Chitosan/collagen/gold nanoparticles/biotin-quat188-chitosan (Bi-QCS-AuNPs@collagen)	Layer-by-layer assembly	-	-	Bi-QCS-AuNPs@collagen overcame the low drug load capacity of AuNPs from 64.675 to 87.46% as well as excellent anti-inflammatory activity in macrophage cell lines (RAW264.7). Moreover, in comparison to free 5-FU, the nanohybrid improved drug activity by 3.3-fold in HeLa cell lines and 6.2-fold in A549 cell lines, respectively.	[168]
Nanoparticles	Voriconazole	Chitosan	Spray-drying	Topical	Skin	The drug loading in NPs ranged from 75% to 90%. Sustained-release profile in rat skin model and exhibited antifungal activity against *C. albicans.*	[169]
Films	Ciprofloxacin	Chitosan/chitosan-depolymerization products	Casting	-	-	Low acetylated and molecular weight CDP-based films exhibited reduced swelling and ciprofloxacin released in a controlled manner for up to 54% in 24 h in a pH-dependent manner.	[170]

**Table 3:** Recent studies focusing on the wound healing applications of chitosan formulation.

Device Type	Model Drug/Drug	Polymer Formulation	Preparation Method	Wound Site	Effects/Results	References
Hydrogels	-	Gallic acid/chitosan	Discharge plasma technology		Traditional DPPH scavenging experiments revealed remarkable antioxidant characteristics. CS-GA formed hydrogels by cross-linking by undergoing oxidation at a physiological state. High cytocompatibility and hemocompatibility were observed in vivo rat skin-layer defects and liver hemorrhagic models (46.6% collagen fiber growth by the 7th day). CS-GA functions at the wound site without the application of prolonged pressure.	[297]
Hydrogels		Catechol-modified chitosan/Oyster peptide microspheres/β-sodium glycerophosphate (β-GP)				[298]
Hydrogels	Amoxicillin, tetracycline, cefuroxime, acetylsalicylic acid	Chitosan/genipin		Ulcer wounds/dermal tissue	Synergistic antibacterial-anti-inflammatory wound healing was observed with ASA-based antibiotic combinations with sustained drug release.	[299]
Hydrogels		Carboxymethyl chitosan/oxidized quaternized guar gum OQGG@CMCS			Excellent antibacterial and hemostatic activity, self-healing in *S. aureus* rat model.	[300]
Electrospun mats		Chitosan/poly-ε-caprolactone fibrous mat/polyurethane foam/ethanolic extract of propolis (EEP)	Electrospinning		PCL/CS-PU/EEP bilayered wound dressing exhibited improved biocompatibility and healing potential both in vitro and in vivo.	[301]
Thermosensitive hydrogel-microparticles based hybrid		Chitosan/β-glycerophosphate thermosensitive hydrogel/decellularized amniotic membrane/polylactic acid microparticles			The hybrid oxygen-generating wound dressing material promoted cell adhesion and growth and was non-cytotoxic, and released oxygen for 7 days.	[302]
Bionanocomposite		Carboxymethyl cellulose/tragacanth gum/silver-titanium nanoparticles	Freeze drying		The wound dressing exhibited porosity between 65–79%, which increased with the addition of silver-TiO_2_. Higher wound dressing weight loss was observed for the highest concentration of AgO/TiO_2_.	[303]
Hydrogels		Chitosan/lignin/polyvinyl alcohol	Freeze thawing		Lignin-based PVA-chitosan hydrogels had good mechanical strength, protein adsorbing capacity, and wound healing with environmental regulation ability.	[304]
Membrane		Chitosan/hyaluronan/phosphatidylcholine dihydroquercetin (Ch/HA/PCDQ)			Ch/HA/PCDQ membranes displayed antibacterial, antioxidant, and anti-inflammatory activities as well as showed biocompatibility. Significantly greater wound healing potency was observed in mouse full thickness wound model.	[305]
Nanoparticles-loaded electrospun nanofibers	OH-CATH30 antibacterial peptide	Chitosan/polyvinyl alcohol	Electrospinning	Skin wounds	NP-30-NFs exhibited antibacterial activity against *E.coli* and *S. aureus* and showed wound healing in mouse skin wounds.	[306]

## Data Availability

Not applicable.

## Abbreviations

5	FU-5-fluorouracil
AC	Adenine-functionalized chitosan
ADSCs	Adipose mesenchymal stem cells
BP	QCS-quaternized chitosan-modified black phosphorus nanosheets
BSA	Bovine serum albumin
CMCS	Carboxymethyl chitosan
CMCTS	Carboxymethyl chitosan
CNC	Cellulose nanocrystals
CNs	Chitosan nanoparticles
COS	Chitosan-oligosaccharides
CPO	Calcium peroxide
CS	Chitosan
CS/DA	Chitosan/dicarboxylic acid
CS-HA	Hydrocaffeic acid-modified chitosan
CUR	Curcumin
DAFM	Decellularized annulus fibrosis matrix
DCs	Dendronized chitosans
DD	Degree of deacetylation
DDS	Drug delivery systems
DTX	Docetaxel
EC	Ethylcellulose
ECM	Extracellular matrix
EPCs	Endothelial progenitor cells
F/C	Fucoidan/chitosan
GA	Glycyrrhetinic acid
GelMA	Gelatin methacryloyl
H/Al-MSN	Aluminum-modified mesoporous silica nanoparticles
HA	Hyaluronic acid
hmCS	Hydrophobically modified chitosan
HPLCs	Human periodontal ligament cells
HUVECs	Human umbilical vein endothelial cells
iSur -	pDNA-survivin shRNA-expressing plasmids
LA	Lactobionic acid
MACS	Micro-channeled alkylated chitosan sponge
MCS	Maleic chitosan
MT NPs	p-mercaptobenzoic acid-embedded N, N, N-trimethyl chitosan nanoparticles
MTT	3-(4,5-dimethylthiazol-2-yl)-2,5-diphenyl tetrazolium bromide
nHA	Nano-hydroxyapatite
NP	Nanoparticle
OEG	Oligoethylene glycol
OQGG@CMCS	Carboxymethyl chitosan/oxidized quaternized guar gum
PAA	Polyacrylic acid
PCADK	Poly (cyclohexane-1, 4-diylacetone dimethylene ketal)
PCL	Poly(-caprolactone)
PEC	Polyelectrolyte complex
PEGDA	Poly(ethylene glycol) diacrylate
PEM	Polyelectrolyte multilayer
PTX	Paclitaxel
RHC-CHI	Recombinant human collagen-chitosan
rhIL-2	Recombinant human interleukin-2
ROS	Reactive oxygen species
SA	Sodium alginate
SAD	Sodium alginate dialdehyde
SCO	Splice correction oligonucleotides
SHED	Stem cells from human exfoliated deciduous teeth
Si-HPMC	Silanized-hydroxypropyl methylcellulose
siRNA	Small interfering RNA
TPEG	Thiol-terminated poly(ethylene glycol)
